# A Phase I Study of Vincristine, Irinotecan, Temozolomide and Bevacizumab (Vitb) in Pediatric Patients with Relapsed Solid Tumors

**DOI:** 10.1371/journal.pone.0068416

**Published:** 2013-07-22

**Authors:** Rajkumar Venkatramani, Marcio Malogolowkin, Tom B. Davidson, William May, Richard Sposto, Leo Mascarenhas

**Affiliations:** 1 Division of Hematology/Oncology, Children’s Hospital Los Angeles, Los Angeles, California, United States of America; 2 Department of Pediatrics, Keck School of Medicine, University of Southern California, Los Angeles, California, United States of America; 3 Department of Pediatrics, Medical College of Wisconsin, Milwaukee, Wisconsin, United States of America; 4 Department of Pediatrics, David Geffen School of Medicine, University of California Los Angeles, Los Angeles, California, United States of America; 5 Department of Preventive Medicine, Keck School of Medicine, University of Southern California, Los Angeles, California, United States of America; Davidoff Center, Israel

## Abstract

**Background:**

To determine the maximum tolerated dose (MTD) and dose limiting toxicity (DLT) of irinotecan administered in combination with vincristine, temozolomide and bevacizumab in children with refractory solid tumors.

**Methods:**

The study design included two dose levels (DL) of irinotecan given intravenously once daily for 5 consecutive days (DL1: 30 mg/m^2^, and DL2: 50 mg/m^2^), combined with vincristine 1.5 mg/m^2^ on days 1 and 8, temozolomide 100 mg/m^2^ on days 1-5, and bevacizumab 15mg/kg on day 1, administered every 21 days for a maximum of 12 cycles.

**Results:**

Thirteen patients were enrolled and 12 were evaluable for toxicity Dose limiting toxicity observed included grade 3 hyperbilirubinemia in 1 of 6 patients on DL1, and grade 3 colitis in 1 of 6 patients on DL2. DL 2 was the determined MTD. A total of 87 cycles were administered. Myelosuppression was mild. Grade 1-2 diarrhea occurred in the majority of cycles with grade 3 diarrhea occurring in only one cycle. Grade 2 hypertension developed in two patients. Severe hemorrhage, intestinal perforation, posterior leukoencephalopathy or growth plate abnormalities were not observed. Objective responses were noted in three Wilms tumor patients and one each of medulloblastoma and hepatocellular carcinoma. Five patients completed all 12 cycles of protocol therapy.

**Conclusions:**

Irinotecan 50 mg/m^2^/day for 5 days was the MTD when combined with vincristine, temozolomide and bevacizumab administered on a 21 day schedule. Encouraging anti-tumor activity was noted.

**Trial Registration:**

ClinicalTrials.gov; NCT00993044; http://clinicaltrials.gov/show/NCT00993044

## Introduction

The combination of irinotecan and temozolomide has shown activity against many solid tumors including neuroblastoma, Ewing sarcoma, and rhabdomyosarcoma [1,2]. There are both preclinical and clinical evidence of synergy between these two agents, and this may be schedule dependent [3,4]. The non-overlapping dose limiting toxicities of these two agents, diarrhea (irinotecan) and myelosuppression (temozolomide) make this combination attractive. In addition, irinotecan and vincristine have shown synergistic activity in patients with rhabdomyosarcoma [5]. Based on preclinical data, irinotecan was initially administered as a protracted regimen (five consecutive days in two weeks) [4,6]. Subsequently, studies have shown that there was no difference in efficacy between irinotecan administered as protracted regimen or as shortened regimen over five days [5,7]. The Children’s Oncology Group (COG) has studied the combination of vincristine, oral irinotecan and temozolomide in the phase I setting, and demonstrated its feasibility and safety [7]. Combining newer targeted agents to this backbone may provide additional anti-tumor activity.

Angiogenesis is the hallmark of tumor development and metastases. Bevacizumab is a humanized monoclonal neutralizing antibody against vascular endothelial growth factor [8]. Bevacizumab is approved in adults for use in colorectal, renal, non-small cell lung cancer and glioblastoma [9–11]. Bevacizumab has shown activity in preclinical models of pediatric cancers [12–14]. Bevacizumab at a dose of 15 mg/kg administered every two weeks was well tolerated in a phase I study conducted by COG [15]. Even though no objective responses were seen in that study, responses were observed when bevacizumab was used in combination with irinotecan in children with low and high-grade glioma [16,17]. Anecdotal reports and case series of combination of bevacizumab, irinotecan and temozolomide have been published, but this combination has not been systematically studied in children [18]. The maximum tolerated dose of irinotecan administered intravenously over five days in combination with temozolomide is yet to be defined. A previous study of irinotecan and temozolomide performed in neuroblastoma patients used a lower threshold for platelets [1]. We conducted a phase I study of escalating doses of irinotecan together with standard doses of vincristine, temozolomide and bevacizumab (VITB) in patients with relapsed or refractory solid tumors.

## Materials and Methods

The protocol for this trial and supporting TREND checklist are available as supporting information; see Checklist S1 and Protocol S1.

### Ethics Statement

This research was approved by the institutional review board at Children’s Hospital, Los Angeles. Written informed consent was obtained from patients. In case of minors, written informed consent was obtained from parents/legal guardians.

### Patient Eligibility

Patients >1 and <21 years of age with histologically confirmed solid tumor without known effective therapy, body weight ≥ 10 kilograms, a Karnofsky or Lansky performance score of > 50%, and with an expected life expectancy of > 8 weeks were eligible. Patients must have recovered from acute toxic effects of prior therapy; and must not have received 1) myelosuppressive therapy within two weeks (four weeks if nitrosourea); 2) biological agent within one week; 3) small-port palliative radiotherapy within 2 weeks; 3) total body, craniospinal or hemi-pelvic radiation within 6 months; 4) autologous stem cell transplant within six months; 5) hematopoietic growth factors within 1 week. Organ function requirements were as follows: peripheral blood neutrophil count (ANC) ≥ 750/µL, platelet count ≥ 75,000/µL (transfusion-independent), Hemoglobin ≥ 8 g/dL; ANC ≥ 500/µL, platelet count ≥ 50,000/µL (transfusion-independent) in patients with bone marrow involvement; normal serum creatinine for age or a glomerular filtration rate of > 70ml/min/m^2^; urine protein creatinine ratio <0.5; left ventricular shortening fraction of ≥ 28% by echocardiogram or ejection fraction of ≥ 55% by MUGA scan; total bilirubin level ≤ 1.5 x upper limit of normal for age, alanine aminotransferase ≤ 5 x upper limit of normal for age. Patients who underwent a major surgical procedure within 28 days or a minor surgical procedure within seven days were ineligible. Patients with a history of deep vein thrombosis within three months, cerebrovascular accident within six months, or with uncontrolled hypertension were excluded. Patients who had received irinotecan or bevacizumab previously were ineligible. Patients who had received temozolomide were ineligible except for patients with central nervous system tumors. Pregnant or breastfeeding females, patients with uncontrolled infection were excluded. The study was registered in ClinicalTrials.gov (NCT00993044).

### Study Design

Each cycle was 21 days and included: vincristine 1.5 mg/m^2^ (2 mg maximum total dose) intravenously on days 1 and 8; bevacizumab 15mg/kg intravenously on day 1 administered over 90 minutes during cycle 1, over 60 minutes in cycle 2 and over 30 minutes from cycle 3 onward provided there was no reaction during infusion; temozolomide 100mg/m^2^ orally on days 1-5; irinotecan intravenously on days 1-5 approximately 60 minutes after temozolomide intake. Patients received granulocyte colony stimulating factor (G-CSF) 5 µg/kg/day subcutaneously starting on day 6 and continued until ANC> 2000/µL, or one dose of PEG filgrastrim 100 µg/kg. Patients received a maximum of 12 cycles in the absence of disease progression.

Irinotecan was administered intravenously on days 1-5 of each 21 day cycle. Two dose levels were planned: dose level 1, 30 mg/m^2^ (maximum 60 mg/day); dose level 2, 50 mg/m^2^ (maximum 100mg/day), with the option of two de-escalation dose levels: dose level -1, 20 mg/m^2^ (maximum 40 mg/day) and dose level 1.5, 40 mg/m^2^ (maximum 80 mg/day). In the event of diarrhea within 12 hours of irinotecan administration, patients received atropine 0.01mg/kg intravenously. If diarrhea occurred after 12 hours, or after administration of atropine, oral loperamide was administered. If the patient developed ≥ grade 3 diarrhea, cefixime or cefpodoxime was started 5 days before administration of irinotecan on subsequent cycles and continued for a minimum of 24 hours after the last dose of irinotecan.

A minimum of three evaluable patients were treated at each dose level. In the absence of dose limiting toxicity (DLT), patients were enrolled at the next dose level. If 1 of 3 patients had a DLT, the cohort was expanded to include 6 patients. If ≥ 2 patients experience DLT, maximum tolerated dose (MTD) was exceeded and further enrollment at that dose level was stopped. The MTD was the highest dose level at which ≤ 1 of 6 patients experienced a DLT. If the MTD was exceeded at the dose level 1, then the subsequent cohort of patients were to be treated at dose level -1. If the MTD was exceeded at the dose level 2, then the subsequent cohort of patients were to be treated at dose level 1.5. Patients with DLT who had no evidence of progressive disease were allowed to continue on protocol therapy at the lower dose level as long as all toxicities returned to baseline.

### Patient Evaluation

A medical history, physical examination, renal and liver function tests and serum electrolytes were obtained prior to study enrollment, weekly during the first cycle of treatment and prior to each cycle thereafter. Complete blood counts (CBC) were obtained prior to study enrollment and twice weekly during each cycle. Coagulation studies and urine protein creatinine ratio were obtained prior to each cycle. Echocardiogram was obtained prior to study enrollment, following cycle 2, and at the end of treatment. Tibial growth plate evaluation was performed by plain radiograph of the right or left knee prior to enrollment. If the growth plate was open, further evaluations of the same knee were performed at the end of cycles 2, 5, 8 and 12. Response Evaluation Criteria in Solid Tumors (RECIST) were used to assess tumor response at the end of cycles 2, 5, 8 and 12. Responses were required to be sustained for a minimum of two consecutive imaging evaluations.

Toxicities were graded according to NCI Common Terminology Criteria for Adverse Events (CTCAE), Version 4.0. Hematological dose limiting toxicity was defined as grade 4 neutropenia or grade 4 thrombocytopenia lasting >14 days or myelosuppression which caused delay in chemotherapy for >14 days. Any grade 3 or grade 4 non-hematological toxicity was considered a dose limiting toxicity with the specific exclusion of 1) grade 3 or 4 nausea and vomiting controlled by anti-emetics; 2) grade 3 transaminase elevation which returned to ≤ grade 1 before next cycle; 3) grade 3 diarrhea lasting < 3 days; 4) grade 3 infection or fever; 5) grade 3 electrolyte abnormalities that improved to ≤ grade 2 within seven days; 6) grade 3 catheter related venous thrombosis; 7) vincristine related neuropathy. In addition, grade 2 arterial thrombosis, and grade 2 pulmonary or CNS hemorrhage were considered dose limiting. Only, toxicities during the first 2 cycles were used to determine the MTD.

## Results

### Patient characteristics

**Figure 1:**
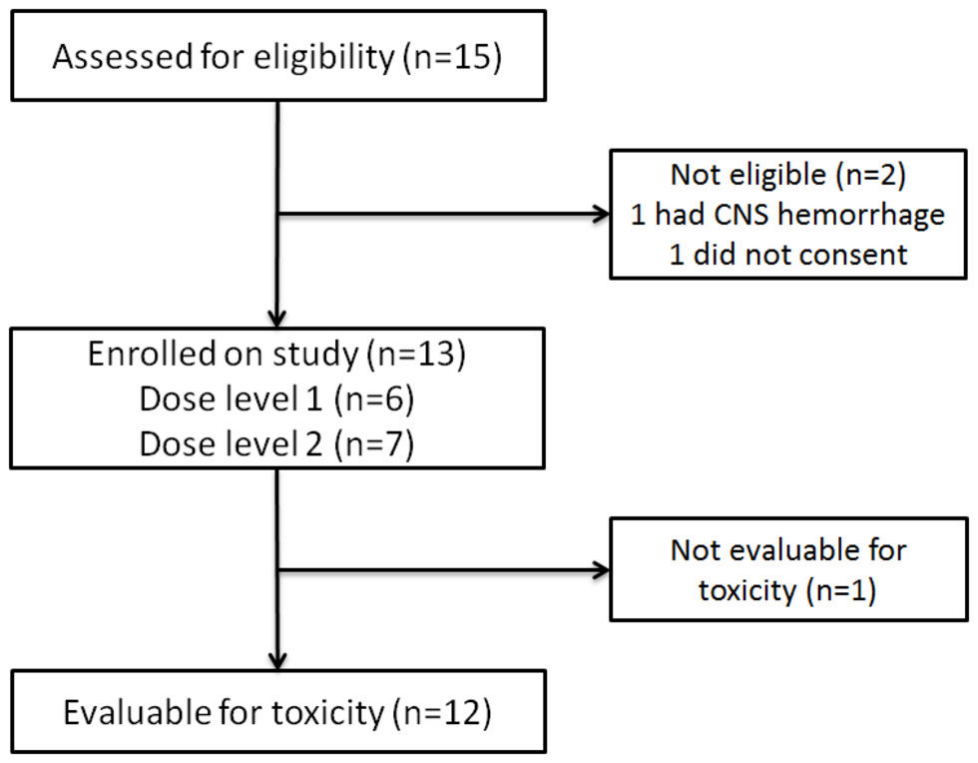
Patient flow diagram.

Thirteen patients were prospectively enrolled between December 2009 and August 2012 (Table 1 Figure 1). One patient developed fever on the second day of cycle 1 and did not receive further protocol therapy. This patient was removed from the study and deemed ineligible for toxicity evaluation since the fever was unrelated to therapy. Twelve eligible patients received 87 cycles of therapy (47 on dose level 1, 40 on dose level 2). Four patients had previously received high-dose chemotherapy followed by autologous bone marrow transplant.

**Table 1:** Patient Characteristics (n=12).

**Category**	**Subcategory**	**No. of Patients (%)**
**Age at enrollment, y**	Median	11
	Range	3.9-19.4
**Gender**	Male	8 (66.7)
	Female	4 (33.3)
**Diagnosis**	Wilms tumor	3 (25)
	Osteosarcoma	2 (16.7)
	Hepatocellular carcinoma	2 (16.7)
	Ewing sarcoma	1 (8.3)
	Medulloblastoma	1 (8.3)
	Liposarcoma	1 (8.3)
	Synovial Sarcoma	1 (8.3)
	Angiosarcoma	1 (8.3)
**Prior therapy**	No. of chemotherapy regimens, Median (range)	2 (1-4)
	Radiotherapy	7
	Autologous bone marrow transplant	4

### Toxicity

The first patient enrolled on dose level 1 had Noonan syndrome and recurrent synovial sarcoma. This patient developed dose limiting hyperbilirubinemia during cycle 1 and was removed from protocol therapy after two cycles due to non-compliance. None of the additional five patients enrolled on dose level 1 developed dose limiting toxicity. Dose limiting colitis developed in one of the first three patients enrolled on dose level 2. Three additional patients enrolled at this level did not have a DLT. Therefore dose level 2 (irinotecan 50 mg/m^2^) was determined to be the MTD. Toxicities which developed during the first two cycles were used to determine the MTD in an attempt to capture possible delayed toxicity due to bevacizumab.

The hematological toxicities are summarized in Table 2. None of the hematological toxicities met the study definition of DLT. Due to the potential for bleeding when bevacizumab is administered, platelet transfusion was administered for platelet count less than 20,000/µL. In spite of this requirement, five patients on this study did not require platelet or packed red blood cell transfusion throughout their therapy (median 5 cycles, total 28 cycles). Therapy was administered in the ambulatory setting, and hospitalization for fever and neutropenia occurred only in three cycles. The frequency of grade 3 or 4 non-hematological toxicity was low (Table 3). Two patients developed grade 2 hypertension; one was transient and related to pain. In the other patient, hypertension developed during cycle 11 and was well controlled with low-dose single agent anti-hypertensive treatment. This patient was able to continue protocol therapy without interruption. Severe proteinuria requiring discontinuing treatment was not observed. Mild (grade 1) abnormalities in coagulation studies were detected in 14 cycles. There were no episodes of thrombosis, and intermittent grade 1 epistaxis was seen in four patients. Growth plate changes were not seen in serial knee radiographs obtained in seven patients (median of 5 cycles) whose growth plate was open at study enrollment. Mild diarrhea (grade 1 and 2) occurred in 60 cycles, and was well controlled with loperamide.

**Table 2:** Hematological Toxicities.

**Toxicity**	**Cycle 1 (N=12)**			**Cycles 2-12 (N=75)**		
	**Grade**			**Grade**		
	**2**	**3**	**4**	**2**	**3**	**4**
Anemia	3	2		28	19	
Leukopenia			2	11	7	2
Neutropenia	1	1	4	5	7	11
Thrombocytopenia		1	2	9	6	8

**Table 3:** Non-hematological Toxicities.

**Toxicity**	**Cycle 1 (N=12)**		**Cycles 2-12 (N=75)**	
	**Grade 3**	**Grade 4**	**Grade 3**	**Grade 4**
ALT	1			
Colitis	1*			1
Diarrhea			1	
Fever Neutropenia	2		1	
Hyperbilirubinemia	1*		1	
Hypoalbuminemia			1	
Hypocalcemia			1	
Hypokalemia	1		1	
Hyponatremia	1		1	
Neuropathy			1	

* Dose limiting toxicity

### Antitumor activity

Complete response was achieved in a patient with Wilms tumor after 8 cycles (Table 4). In another patient with relapsed Wilms tumor, a lung nodule persisted despite a dramatic response initially. This nodule was resected after 8 cycles, and did not have histological evidence of viable tumor. Therefore, this patient was determined to have a complete response. Partial responses were seen in patients with Wilms tumor, medulloblastoma and hepatocellular carcinoma. The patient with medulloblastoma was removed from protocol therapy by the primary oncologist after 4 cycles to administer radiation therapy. One patient was removed from protocol therapy after cycle 1 due to progressive disease. Another patient with an angiosarcoma of the heart and a 8 cm x 3 cm chest wall mass showed a dramatic response to the first two cycles with greater than 50% reduction in tumor size on imaging. The start of his third cycle was delayed due to fever by one week. He developed progressive disease during this period and was removed from protocol therapy. Five patients completed all 12 cycles of chemotherapy.

**Table 4:** Dose-limiting Toxicity and Response.

**Tumor Type**	**Dose Level**	**No. of courses**	**Best Response**	**Dose limiting toxicity**	**Reason for coming off protocol therapy**
Synovial Sarcoma	1	2	SD	Hyperbilirubinemia	Toxicity/non compliance
Osteosarcoma	1	5	SD		PD
Hepatocellular carcinoma	1	12	SD		End of therapy
Liposarcoma	1	12	SD		End of therapy
Medulloblastoma	1	4	PR		Physician preference
Wilms Tumor	1	12	CR		End of therapy
Hepatocellular carcinoma	2	12	PR		End of therapy
Angiosarcoma	2	2	PD		PD
Osteosarcoma	2	1	PD	Colitis	PD
Wilms Tumor	2	8	PR		PD
Wilms Tumor	2	12	CR		End of therapy
Ewing Sarcoma	2	5	SD		PD

SD, stable disease; PD, progressive disease; PR, partial response; CR, complete response

## Discussion

Irinotecan has been administered using various schedules in both adults and children [6,19–22]. Preclinical studies in pediatric tumors by Houghton et al, showed that a protracted schedule of irinotecan given daily for 5 days per week for 2 consecutive weeks resulted in greater response rates when compared to the same dose administered over 5 days [23]. Based on this, irinotecan was initially administered on a protracted schedule of 5 consecutive days for 2 weeks in pediatric studies [4,6]. A subsequent randomized phase II study of protracted versus 5 day schedule of irinotecan did not show any difference in response rates in rhabdomyosarcoma patients [5]. Another trial comparing the two regimens of oral irinotecan given with vincristine and temozolomide reported higher frequency of dose limiting toxicity in the protracted regimen [7]. Since the 5 day schedule is more convenient for patients, we used this schedule in our trial. The MTD for irinotecan administered as a single agent in a 5 day regimen ranges from 39-50 mg/m^2^ depending on the number of previous treatment regimens received [19]. Myelosuppression was dose limiting in heavily pretreated patients while diarrhea was dose limiting in less heavily pretreated patients. Irinotecan 50mg/m^2^ and temozolomide 150 mg/m^2^ administered over 5 days every 3-4 weeks has been studied in neuroblastoma patients, but this study used a lower platelet count threshold of 30,000/µL for administering subsequent cycles. Therefore, we decided to study escalating dose levels of irinotecan.

Overall this regimen was tolerated well. There was no delay in therapy due to hematological toxicity. Similar to other studies with this backbone, the number of patients requiring platelet or blood transfusions was low [4]. Based on our experience in this study, routine use of myeloid growth factors may not be needed with this regimen. Even though we did not use prophylactic antibiotics, diarrhea was well controlled with loperamide, and only one patient developed grade 3 diarrhea. Majority of grade 3 and 4 toxicities described in Table 3 occurred in one patient with Noonan syndrome. We do not know if Noonan syndrome predisposed this patient to have more toxicity. The dose limiting hyperbilirubinemia is most likely attributable to irinotecan. Hyperbilirubinemia has been reported with the use of irinotecan in both single agent and combination pediatric studies [7,19].

Known serious adverse effects of bevacizumab including severe hemorrhage, gastrointestinal perforation, arterial thromboembolism, posterior leukoencephalopathy and cardiac side effects were not seen. The number of cycles administered in this study may have been too few to detect these rare side effects that are reported in adult studies. Central nervous system hemorrhage, and transient leukoencephalopathy have been reported in children who received bevacizumab [16,24]. The patient who developed hypertension requiring antihypertensive treatment in our study, had a history of bilateral nephron sparing surgery, which may have contributed to developing hypertension. Due to reversible physeal dysplasia seen in juvenile monkeys following bevacizumab administration [25], we performed serial imaging of growth plates in seven patients who had open growth plates. We did not detect any growth plate expansion.

Even though this study was performed primarily to study toxicity, the antitumor activity of this combination is encouraging. Five of the 12 patients (42%) had objective responses. Two other patients had stable disease through 12 cycles. This combination showed significant activity in all 3 patients enrolled with relapsed Wilms tumor. All three patients were heavily pretreated and had a history of previous autologous bone marrow transplant and lung irradiation. This was unexpected as 17 patients with Wilms tumor who had received either single agent irinotecan or irinotecan and temozolomide in previous studies did not show a response [4,19–21]. Blockade of vascular endothelial growth factor has been shown to cause regression of Wilms tumor in preclinical studies [26,27]. Addition of bevacizumab to the chemotherapy backbone may explain the activity observed in Wilms tumor in our study.

The MTD was irinotecan 50 mg/m^2^ on days 1-5 administered with vincristine 1.5 mg/m^2^ on days 1 and 8, temozolomide 100 mg/m^2^ and, bevacizumab 15mg/kg on day 1 every 21 days. This combination was tolerable and showed significant antitumor activity. This study supports additional investigation of this combination, particularly in patients with Wilms tumor. Our study can also serve as a template for adding other targeted therapies to the vincristine, irinotecan and temozolomide chemotherapy backbone.

## Supporting Information

Checklist S1 TREND checklist for nonrandomized controlled trails. (DOC)
(PDF)

Protocol S1 Study Protocol. (DOC)
(PDF)
