# Supplementary material for: A Phase I Study of Vincristine, Irinotecan, Temozolomide and Bevacizumab (Vitb) in Pediatric Patients with Relapsed Solid Tumors
Source: PLoS One. 2013 Jul 22;8(7):e68416. doi: 10.1371/journal.pone.0068416 (PMC3718768; doi:10.1371/journal.pone.0068416)
Supplement: Protocol S1 — (DOC) (PDF) [file pone.0068416.s002.pdf]

Activated: 09/15/2009 Date of Template:07/02/2010 Amendment 2

A PHASE I STUDY OF VINCRISTINE, ESCALATING DOSES OF IRINOTECAN, TEMOZOLOMIDE AND BEVACIZUMAB (VIT-B) IN PEDIATRIC AND ADOLESCENT PATIENTS WITH RECURRENT OR REFRACTORY SOLID TUMORS OF NON-HEMATOPOIETIC ORIGIN.

THIS PROTOCOL IS FOR RESEARCH PURPOSES ONLY, AND SHOULD NOT BE COPIED, REDISTRIBUTED OR USED FOR ANY OTHER PURPOSE.

THE PROCEDURES IN THIS PROTOCOL ARE INTENDED ONLY FOR USE BY CLINICAL ONCOLOGISTS IN CAREFULLY STRUCTURED SETTINGS, AND MAY NOT PROVE TO BE MORE EFFECTIVE THAN STANDARD TREATMENT. A RESPONSIBLE INVESTIGATOR ASSOCIATED WITH THIS CLINICAL TRIAL SHOULD BE CONSULTED BEFORE USING OR ATTEMPTING ANY PROCEDURE IN THIS PROTOCOL.

PRINICIPAL INVESTIGATOR:

Rajkumar Venkatramani, M.D.
Childrens Hospital Los Angeles
Division of Hematology-Oncology
4650, Sunset Boulevard, Mailstop # 54
Los Angeles, California 90027-6016
Phone: 323- 361-2121
Fax: 323-361-7128
rvenkatramani@chla.usc.edu

CO-PRINCIPAL INVESTIGATOR AND FACULTY MENTOR:

Leo Mascarenhas, M.D.
Childrens Hospital Los Angeles
Division of Hematology-Oncology
4650, Sunset Boulevard, Mailstop # 54
Los Angeles, California 90027-6016
Phone: 323- 361-2529
Fax: 323-361-8174
lmascarenhas@chla.usc.edu

Abstract: 4
Experimental Design Schema: 4
1.0 Goals and Objectives (Scientific Aims) 5
1.1 Primary Aims: 5
1.2 Secondary Aim: 5
2.0 Background 5
2.1 Introduction: 5
2.2.2 Rationale for the use of escalating doses of Irinotecan 6
2.2.3 Rationale for the Use of Bevacizumab: 8
2.2.4 Rationale for Combination of Vincristine, Irinotecan, Temozolomide, and Bevacizumab (VIT-B) 9
3.0 Screening and Study Enrollment Procedures 11
4.0 Patient Eligibility: 12
4.1 Inclusion Criteria: 12
4.2 Exclusion Criteria 15
5.0 Treatment Overview: 16
5.4 Dose Escalation 18
5.5 Definitions of Dose-Limiting Toxicity 19
6.0 Dose Modifications for Toxicity: 21
6.1 Dose Modifications/Delays Guidelines for Bevacizumab 21
6.2 Other Modifications 23
7.0 Supportive Care: 24
8.0 Required Observations/Material and Data to Be Accessioned 27
9.0 Agent Information 28
9.1 Vincristine Sulfate 28
9.2 Irinotecan 29
9.3 Temozolomide 31
9.4 Bevacizumab 33
9.5 Myeloid Growth Factors 35
10.0 Criteria for Removal from Protocol Therapy and Off Study Criteria 37
11.0 Statistical Considerations 37
12.0 Evaluation Criteria 38
13.0 Adverse Event Reporting Requirements: 41
14.0 Records and Reporting 44
15.0 Data and Safety Monitoring Plan: 44
16.0 References: 45
APPENDIX I: Performance Status Scales/Scores 49
APPENDIX II: Temozolomide Dosing Table-Starting Dose 50
APPENDIX III: Instructions for Administration of Temozolomide 51

Study Committee:

PRINCIPAL INVESTIGATOR:
Rajkumar Venkatramani, M.D.
Childrens Hospital Los Angeles
Division of Hematology-Oncology
4650, Sunset Boulevard, Mailstop # 54
Los Angeles, California 90027-6016
Phone: 323- 361-2121
Fax: 323-361-7128
rvenkatramani@chla.usc.edu

CO-PRINCIPAL INVESTIGATOR:
Leo Mascarenhas, M.D.
Childrens Hospital Los Angeles
Division of Hematology-Oncology
4650, Sunset Boulevard, Mailstop # 54
Los Angeles, California 90027-6016
Phone: 323- 361-2529
Fax: 323-361-8174
lmascarenhas@chla.usc.edu

Marcio Malogolowkin, M.D.
Childrens Hospital Los Angeles
Division of Hematology-Oncology
4650, Sunset Boulevard, Mailstop # 54
Los Angeles, California 90027-6016
Phone: 323- 361-4514
Fax: 323-361-8174
mmalogolowkin@chla.usc.edu

Girish Dhall, M.D.
Childrens Hospital Los Angeles
Division of Hematology-Oncology
4650, Sunset Boulevard, Mailstop # 54
Los Angeles, California 90027-6016
Phone: 323- 361-8589
Fax: 323-361-8165
gdhall@chla.usc.edu

Jonathan Finlay, MB., ChB
Childrens Hospital Los Angeles
Division of Hematology-Oncology
4650, Sunset Boulevard, Mailstop # 54
Los Angeles, California 90027-6016
Phone: 323- 361-8147
Fax: 323-361-8165
jfinlay@chla.usc.edu

Richard Sposto, Ph.D.
Childrens Hospital Los Angeles
Division of Hematology-Oncology
4650, Sunset Boulevard, Mailstop # 54
Los Angeles, California 90027-6016
Phone: 323- 361-8582
Fax: 323-361-7128
rsposto@chla.usc.edu

Teresa Rushing, Pharm.D
Childrens Hospital Los Angeles
Division of Hematology-Oncology
4650, Sunset Boulevard, Mailstop # 54
Los Angeles, California 90027-6016
Phone: 323- 361-4791
Fax: 323-361-7128
trushing@chla.usc.edu

Jennifer Harrington, BSN
Childrens Hospital Los Angeles
Division of Hematology-Oncology
4650, Sunset Boulevard, Mailstop # 54
Los Angeles, California 90027-6016
Phone: 323- 361-7319
Fax: 323-361-7128
jharrington@chla.usc.edu

Abstract:

Vincristine, Irinotecan and Temozolomide have been used in various combinations to treat pediatric solid tumors. These combinations, in general have been well tolerated. Outcomes for patients with solid tumors who have been treated with various cytotoxic agents have reached a plateau over the last two decades (http://seer.cancer.gov/ csr/1975_2006/results_merged/ sect_28_childhood_cancer.pdf), with similar responses in various resistant cancers. Incorporation of newer agents with different mechanisms of action with cytotoxic chemotherapy may improve response rates and outcomes. Recently several agents that target similar pathways in a broad range of cancers have become available. Only few of them have been tested in the pediatric population. Bevacizumab, an inhibitor of VEGF is one such agent. The novel combination of Vincristine, Irinotecan, Temozolomide and Bevacizumab with their unique mechanisms of action and non-overlapping dose limiting toxicity could be useful in refractory solid tumors. This phase I study is designed to determine the maximum tolerated dose of Irinotecan given intravenous for 5 days every 3 weeks in combination with fixed doses of Vincristine, Temozolomide and Bevacizumab (VIT-B).

Experimental Design Schema:

4 Dose Levels:

Dose Level 1	Irinotecan 30 mg/m^2/day IV on day 1,2,3,4 and 5 (maximum dose 60 mg/day)<br>Vincristine 1.5mg/m^2 (2mg max dose) IV on day 1,8<br>Temozolomide 100 mg/m^2 PO on day 1,2,3,4 and 5<br>Bevacizumab 15mg/kg IV on day 1
Dose Level 1.5*	Irinotecan 40 mg/m^2/day IV on day 1,2,3,4 and 5 (maximum dose 80 mg/day)<br>Vincristine 1.5mg/m^2 (2mg max dose) IV on day 1,8<br>Temozolomide 100 mg/m^2 PO on day 1,2,3,4 and 5<br>Bevacizumab 15mg/kg IV on day 1
Dose Level 2	Irinotecan 50 mg/m^2/day IV on day 1,2,3,4 and 5 (maximum dose 100mg/day)<br>Vincristine 1.5mg/m^2 (2mg max dose) IV on day 1,8<br>Temozolomide 100 mg/m^2 PO on day 1,2,3,4 and 5<br>Bevacizumab 15mg/kg IV on day 1
Dose Level -1	Irinotecan 20 mg/m^2/day IV on day 1,2,3,4 and 5 (maximum dose 40 mg/day)<br>Vincristine 1.5mg/m^2 (2mg max dose) IV on day 1,8<br>Temozolomide 100 mg/m^2 PO on day 1,2,3,4 and 5<br>Bevacizumab 15mg/kg IV on day 1

1. Cycles will be repeated every 21 days

2. Filgrastim 5 mcg/kg SQ once daily, starting 24-48 hrs after the last dose of Irinotecan until ANC> 2000/cmm following nadir or PEG Filgrastim 100mcg/kg SQ X 1 dose 24-48 hrs following last dose of Irinotecan in each cycle. If filgrastrim or PEG filgrastrim is not given 24-48 hrs after the last dose of irinotecan, filgrastrim must be started if the ANC<1000/cmm at anytime during the cycle and continued until the ANC>2000/cmm.
3. Maximum number of cycles = 12

*** Dose escalation will proceed from dose level 1 to dose level 2 and de-escalate to dose level 1.5 if DLT is observed on dose level 2**

1.0 Goals and Objectives (Scientific Aims)

1.1 Primary Aims:

a. To determine the maximum tolerated dose of Irinotecan in combination with fixed doses of Vincristine, Temozolomide and Bevacizumab and recommend a Phase II dose of the combination.
b. To determine the rate of dose limiting toxicities of the combination of Vincristine, Irinotecan, Temozolomide and Bevacizumab (VIT-B).

1.2 Secondary Aim:

To preliminarily define the anti-tumor activity of the combination of, Vincristine, Irinotecan, Temozolomide and Bevacizumab within the confines of a Phase I study.

2.0 Background

2.1 Introduction:

Over the past several decades, significant improvements in the outcome of children with cancer have been documented. However, there are still some patients with poor prognoses, especially those with metastatic sarcomas such as alveolar rhabdomyosarcoma, osteosarcoma, Ewing sarcoma, hepatocellular carcinoma, high-risk neuroblastoma, and certain brain tumors. Additionally, the outlook for most patients who have recurrent disease is dismal. It is because of the many years of life remaining for those pediatric patients who are cured of their malignancy, limiting toxicity and long-term morbidity are also key factors in the treatment of children with cancer. Recent advances in cancer therapy including discovery of various cancer pathways and a plethora of molecular targets have led to introduction of newer anticancer drugs and their combinations. We would like to test one such combination (Vincristine, Irinotecan, Temozolomide and Bevacizumab) in this study.

We hypothesize that:

- The combination of Vincristine, Irinotecan, Temozolomide and Bevacizumab will be tolerable.

- The combination of Vincristine, Irinotecan, Temozolomide and Bevacizumab will have a superior response rate in target cancers (Phase II)

2.2 Rationale:

2.2.1Mechanism of action of drugs in the combination:

Vincristine is a vinca alkaloid that inhibits microtubule formation, arresting mitosis in metaphase.[1] Irinotecan is a camptothecin pro-drug that is de-esterified to the more potent topoisomerase I inhibitor SN-38. Temozolomide is an oral methylating agent. Bevacizumab is a humanized monoclonal neutralizing antibody binding all 5 isoforms of human VEGF.[2] The combination of these 4 drugs which have different mechanisms of action and may be helpful in overcoming tumor resistance and improve outcomes.

2.2.2 Rationale for the use of escalating doses of Irinotecan:

Irinotecan (CPT-11; 7-ethyl-10-(4-[1-piperidino]-1-piperidino) carbonyloxy-camptothecin) is a water soluble analogue of camptothecin. Camptothecins interact specifically with the enzyme topoisomerase I which relieves torsional strain in DNA by inducing reversible single-strand breaks. Irinotecan and its active metabolite SN-38 bind to the topoisomerase I-DNA complex and prevent religation of these single-strand breaks. Current research suggests that the cytotoxicity of irinotecan is due to double-strand DNA damage produced during DNA synthesis when replication enzymes interact with the ternary complex formed by topoisomerase I, DNA, and either irinotecan or SN-38.[3]

Cytotoxicity of camptothecin mainly occurs in S-phase. Cells in S-phase are up to 1000-fold more sensitive then cells in G1- or G2 phase, during brief exposures to the drug. These data predict better antitumor activity with prolonged drug exposure, a phenomenon that has been observed in preclinical models.[4]

Several clinical studies have utilized Irinotecan alone or in combination with Vincristine and/or Temozolomide. (See table below)

Single agent studies with Irinotecan:

Authors	Schedule	MTD (maximum tolerated dose)	DLT (dose limiting toxicity)
**Vassal et al.[5] (2003)**	**120-720 mg/m^2 on day 1 every 21 days**	**600mg/m^2**	**Diarrhea and leukopenia**
**Furman et al[6] (1999)**	**20mg/m^2 (initial dose) (qdx5)x2 every 21 days**	**20mg/m^2**	**diarrhea**
**Hirota et al [7] (2001)**	**100mg/m^2/day(initial dose) X3 every 21 days**	**180 mg/m^2**	**diarrhea**
**Mugishima et al[8] (2002)**	**50mg/m^2/day(initial dose) X3 every 25 days**	**160-180mg/m^2**	**Diarrhea and myelosuppression**
**Blaney et al[9] (2001)**	**30mg/m^2/day(initial dose) x5 every 21 days**	**39mg/m^2-heavily treated patients<br>50mg/m^2- less heavily treated patients**	**Myelosuppression – heavily pretreated patients<br>Diarrhea-less heavily pretreated patients**
**Turner et al[10]**	**125mg/m^2/week x 4 every**	**Phase II**	**myelosuppression**

**(2001) Phase II**	**6 weeks**		
**Bomgaars et al[11] (2007) Phase II**	**50mg/m^2/day x5 every 21 days**	**Phase II**	**Diarrhea and leukopenia**

Combination studies:

Authors	Schedule	MTD	DLT
**Wagner et al.[12] (2004)**	**Irinotecan 10mg/m^2(initial dose) (qdx5)x2 every 21 days**<br><br>**Temozolomide 100 mg/m2 X 5d**	**10mg/m^2**	**Diarrhea and neutropenia**
**Wagner et al.[13] (2007)**	**Irinotecan 10mg/m2(initial dose) (qdx5)x2 every 21 days**<br><br>**Temozolomide 100 mg/m^2 X 5d**	**10-20mg/m^2**	**Diarrhea**
**Packer et al.[14] (2009)**	**Irinotecan 125mg/m^2 every 2 weeks**<br><br>**Bevacizumab 10mg/kg every 2 week**	**125mg/m^2**	**Transient leukoencephalopathy, proteinuria**
**Wagner et al.[15] (2009)**	**Irinotecan 30mg/m^2 (initial dose) PO d1-5 and d8-12 every 21 days**<br><br>**Temozolomide 100mg/m^2/day PO d1-5**	**60mg/m^2**<br><br>**75mg/m^2**	**Neutropenia and thrombocytopenia**
**Pappo et al[16] (2007)**	**Irinotecan 20mg/m^2 (Qdx5)X2**<br><br>**Vincristine 1.5mg/m^2 weeks 0,1,3,4**	**Phase II**	**Abdominal pain, diarrhea**

To summarize, Irinotecan has been used in pediatric studies in various different doses and schedules. Because the shorter schedule of Irinotecan is likely to be a more appealing regimen to patients and families, we propose to use the daily x 5 schedule for irinotecan. This schedule (Irinotecan 50 mg/m^2/day iv daily x 5 with weekly Vincristine) was studied in patients with recurrent rhabdomyosarcoma[17] and was determined to be as well tolerated and as effective as the protracted (20 mg/m^2/day daily x 5 x 2 with weekly Vincristine) schedule. Bomgaars et al showed that Irinotecan at 50 mg/m^2/day x 5 days every 3 weeks was well tolerated in patients with refractory solid tumors.[11]

In a recently published abstract Wagner et al[18] compared 2 different schedules of oral

irinotecan (d x5 x 2 vs. d x 5x 1) in combination with Vincristine and Temozolomide in children with refractory solid tumors. They showed that d x 5x 1 schedule was well tolerated at oral Irinotecan dose of 90 mg/m2/day and no DLT was seen.

Blaney et al[9] showed that the MTD of Irinotecan, when given as single agent, was 39 mg/m2/day x 5 days for heavily pretreated patients and 50 mg/m2/day x 5 days for less heavily pretreated patients. Kushner et al[19] reported that 50 mg/m^2 /day x 5 of Irinotecan with Temozolomide 150 mg/m2/day x 5 days was tolerated by patients with recurrent neuroblastoma. However, the hematologic parameters for dosing on this study were lower than what is generally accepted as standard. Based on the available data, the MTD for Irinotecan when given in combination with other drugs is yet to be firmly established. Therefore we propose to test Irinotecan at a dose of 30 mg/m^2/day x 5 days and subsequently escalate the dose to 50 mg/m2/day x 5 days if tolerated.

2.2.3 Rationale for the Use of Bevacizumab:

Angiogenesis is the hallmark of tumor development and metastases. Vascular endothelial growth factor (VEGF) is the most extensively studied proangiogenic factor. Tumor cells up regulate the expression of VEGF when they grow in response to decreased levels of oxygen and nutrients and the accumulation of metabolic waste. The binding of VEGF to its receptor VEGFR-2 is a critical step that stimulates endothelial cell proliferation, migration, tube formation, and capillary sprouting.[20]

Bevacizumab (Avastin ®)

Bevacizumab is a humanized monoclonal neutralizing antibody binding all 5 isoforms of human VEGF[2]. It has been shown to bind to VEGF-A with high affinity and neutralize its activity.[21] Inhibition of angiogenesis has resulted in reduced tumor growth in many ex vivo models of pediatric and adult malignancies.[22-27] This effect has also been independently demonstrated in RMS xenograft tumors.[26] Animal toxicology studies of Bevacizumab in cynomolgus monkeys demonstrated no acute toxicities but evidence of physeal dysplasia, delayed wound healing and proteinuria were noted.[28]

Administration of Bevacizumab as a single agent to humans with metastatic clear cell carcinoma of the kidney resulted in a significantly longer time to progression compared with placebo.[29] Furthermore, Bevacizumab has been shown to have a direct anti-vascular effect on tumors in patients with adenocarcinoma of the rectum.[30] Although anti-VEGF-A therapy alone appears to have a direct anti-tumor effect, there is evidence from a variety of in vivo models that the combination of anti-VEGF-A therapy and chemotherapy or radiotherapy has greater anti-tumor effects than either therapy alone[31,32] Importantly, a recently reported trial in which adults with metastatic colon cancer were randomized to receive irinotecan, fluorouracil, and leucovorin alone or in combination with Bevacizumab showed that those treated with Bevacizumab plus chemotherapy had a significantly better overall and progression-free survival[33] Toxicities noted in adults are hypertension, epistaxis, proteinuria and infusional reactions. Serious but uncommon events, which appear to be agent specific, include intratumoral bleeding, arterial and deep venous thrombosis, gastrointestinal perforation, nasal septum perforation, impaired

wound healing and reversible posterior encephalopathy syndrome[34,35] From these studies, the overall risk for Grade III/IV toxicities is the 25% range, with Shah et al reporting Grade III hypertension in 28% and thromboembolic events in 25%. These data suggest that Bevacizumab alone and in combination with conventional chemotherapy, has anti-tumor effects and despite some toxicity can safely be given in combination with several standard chemotherapy regimens in adults. These data also suggest that Bevacizumab may be an effective therapeutic approach in multiple types of human cancer in which VEGF-dependent angiogenesis plays an important role in tumor cell survival.

ADVL0314 was a Phase I study of Bevacizumab conducted by COG for children with refractory solid tumors.[36] This single agent study evaluated three dose levels but did not escalate to a maximum tolerated dose (MTD). Side effects were limited to mild infusional reactions, fever, rash, epistaxis, lymphopenia, and proteinuria, none of which necessitated dose modification. There were no episodes of severe hemorrhage or thrombosis. The maximum dose level of 15 mg/kg administered every 2 weeks over 30-90 minutes will be the recommended dose and schedule for future studies of Bevacizumab in children. There were no objective responses on ADVL0314, but 5 of 18 patients with evaluable or measurable disease experienced prolonged disease stabilization (> 3 months), one of which received 16 cycles of therapy prior to progression. All 5 of these patients had a diagnosis of bone or soft tissue sarcoma. Results of a Phase III AVAIL trial in Non Small Cell Lung Cancer that used Bevacizumab combined with gemcitabine and cisplatin were recently published.[37] This trial supports the use of Bevacizumab administered in a once every three week schedule when administered in combination with cytotoxic chemotherapy.

2.2.4 Rationale for Combination of Vincristine, Irinotecan, Temozolomide, and Bevacizumab (VIT-B)

The ideal combination chemotherapy is the one with additive clinical benefit and non additive adverse effects. The dose limiting toxicity of Vincristine (neurotoxicity) Irinotecan (diarrhea, myelosuppression), Temozolomide (myelosuppression), and Bevacizumab (none) combination fits that description. Various combinations of the above agents have been tested in pediatric and adult settings, though not all of them together in the same setting.

Wagner et al did a phase I study to estimate the maximum tolerated dose (MTD) of temozolomide and irinotecan given on a protracted schedule in 28-day courses to pediatric patients with refractory solid tumors.[12] Twelve heavily pretreated patients received 56 courses of oral Temozolomide at 100 mg/m^2/day for 5 days combined with i.v. irinotecan given daily for 5 days for 2 consecutive weeks at either 10 mg/m^2/day (n = 6) or 15 mg/m2/day (n=6). 2 patients experienced DLT (diarrhea, neutropenia) at the higher dose level.

Packer et al treated 10 children with multiply recurrent low grade gliomas with Bevacizumab (10 mg/kg every 2 weeks) and irinotecan (125 mg/m^2 every 2 weeks).[14] Clinical improvements were noted in seven and the dose limiting toxicity included

transient leukoencephalopathy (1) and grade 3 proteinuria (1). The same combination (with a different dosing regimen) was used by Vredenburgh et al in adults with glioblastoma multiforme.[38] Twenty of the 35 patients (57%; 95% CI, 39% to 74%) had at least a partial response. One patient developed a CNS hemorrhage, which occurred in his 10th cycle. Four patients developed thromboembolic complications (deep venous thrombosis and/or pulmonary emboli).

The Children's Oncology Group compared the combination of two cycles of irinotecan (20 mg/m^2 daily for 5 days for 2 weeks) and weekly Vincristine with irinotecan alone in 21 newly diagnosed patients with metastatic rhabdomyosarcoma or undifferentiated sarcoma.[16] The vincristine and irinotecan combination was well tolerated and gastrointestinal toxicity was the main side effect. The combination showed a lower rate of progressive disease (8% vs. 42%).

Bevacizumab in combination with other chemotherapeutic agents has shown efficacy in adults with colorectal cancer, non small cell lung cancer and breast cancer.[33,39-43]There are no reported studies with Bevacizumab that has shown efficacy in pediatric solid tumors to date. This study will combine bevacizumab in combination with cytotoxic chemotherapy and may provide some information on the anti-tumor activity of this combination in the population studied.

3.0 Screening and Study Enrollment Procedures

Physicians who wish to enroll patients should contact the research coordinator to determine if the study is currently open for accrual.

3.0.1 IRB Approval
Local IRB approval should be confirmed prior to enrolling a patient.

3.0.2 Screening Procedures
Diagnostic or laboratory studies performed exclusively to determine eligibility for this trial must only be done after obtaining written informed consent. Studies or procedures that were performed for clinical indications (not exclusively to determine eligibility) may be used for baseline values even if the studies were done before informed consent was obtained.

3.0.3 Informed Consent/Assent
The investigational nature and objectives of the trial, the procedures and treatments involved and their attendant risks and discomforts, and potential alternative therapies will be carefully explained to the patient or the patient's parents or guardian if the patient is a child, and a signed informed consent and assent will be obtained according to institutional guidelines.

3.0.4 Reservation and Contact Requirements
Before enrolling a patient on study, a reservation must be made with the research coordinator at (323) 669-7148. The principal investigator or co-investigators will be contacted. Patients must be enrolled within 5 days of making a reservation.

3.0.5 Patient Registration
The patient will be assigned a unique study identification number once the patient has been enrolled on study.

3.0.6 Eligibility Checklist
Before the patient can be enrolled, the responsible attending physician must sign and date the completed eligibility checklist.

3.0.7 Institutional Pathology Report
At the time of enrollment, the institutional pathology report for the diagnosis under which the patient is being enrolled must be faxed to the research coordinator.

3.0.8 Study Enrollment
Patients may be enrolled on the study once all eligibility requirements for the study have been met. Treatment must start within 5 calendar days of enrollment. **Patients must not receive any therapy as defined/determined by this protocol prior to enrollment and assignment of dose level.**

3.0.9 Dose Assignment

The dose level will be assigned by the Clinical Trials Office (CTO), confirmed by the statistician and Principal Investigator and then communicated to the patient's physician and Principal Investigator in writing by the research coordinator.

4.0 Patient Eligibility:

The eligibility criteria listed below are interpreted literally and cannot be waived.

4.1 Inclusion Criteria:

Patient Status to be confirmed:

Tests to determine eligibility should be performed within one week before enrollment except for disease staging scans that can be performed within two weeks before study enrollment.

Age: Patients must be $\geq$ 12 months and $<$ 21 years of age at the time of study entry.

Weight: Patient must be more than or equal to 10 Kilograms.

Histological Diagnosis: Patients must have had histological verification of the malignancy at some time prior to study entry. All solid tumors are eligible with the exclusion of lymphomas. For patients with neuroblastoma, diagnosis based on elevated catecholamines in the urine and tumor cells on bone marrow aspirates/biopsies is acceptable. For patients with germ cell tumors, diagnosis based on elevated tumor markers (serum alpha fetoprotein and/or serum beta human chorionic gonadotropin) and radiographic evidence of disease is acceptable. Patients diagnosed with diffuse brain stem gliomas by radiographic images alone will be eligible.

Disease Status: Disease must have failed standard therapy (therapies) or be a disease for which no standard therapy exists. Patient with stable disease on other therapies are not eligible.

Performance Level: Karnofsky $\geq$ 50% for patients $\geq$16 years of age and Lansky $\geq$ 50 for children $<$ 16 years of age (Appendix I).

Life Expectancy: Must be $\geq$ 8 weeks.

4.1.1 Prior Therapy:

Patients must have fully recovered from the acute toxic effects of all prior chemotherapy, immunotherapy, or radiotherapy before enrolling on this study:

a. Myelosuppressive chemotherapy: Must not have received within 2 weeks of enrollment on this study (4 weeks if prior nitrosourea).
b. Biologic (anti-neoplastic agent): At least 7 days since the completion of therapy with a biologic agent.
c. XRT: ≥ 2 wks for local palliative XRT (small port); ≥ 6 months must have elapsed if prior craniospinal XRT or if ≥ 50% radiation of pelvis; ≥ 6 wks must have elapsed if other substantial BM radiation.
d. Stem Cell Transplant (SCT): At least 6 months must have elapsed following autologous stem cell transplant. Patients who received an allogeneic transplant are not eligible for this study.
e. Peripheral Blood Stem Cell Infusion:(PBSC) At least 6 months should have elapsed following PBSC infusion for non brain tumor patients.

Patients must have recovered from any surgical procedure before enrolling on this study:
a. Patients with a documented, chronic non-healing wound, ulcer, or significant trauma injury (those with bone fractures, including pathological fractures, or requiring surgical intervention) within 28 days prior to beginning therapy should be excluded.
b. Minor surgical procedures (biopsies) for limited purposes of tissue retrieval will be allowed. Minor procedures include indwelling IV catheter placement and needle biopsy for diagnostic purposes.
    1. For minor surgeries, patients should not receive the first planned dose of Bevacizumab until 7 days have elapsed or the wound is healed, whichever is later.
    2. For procedures such as the placement of an indwelling IV catheter, it is recommended that Bevacizumab not be given for at least 24 hours after the procedure.
c. Patients who have had a major surgical procedure (such as laparotomy, thoracotomy open biopsy or resection of tumor) can only be enrolled on study > 28 days from such procedure.

Patients with unhealed wounds, bone fractures of other bone complications are NOT ELIGIBLE for this study.

4.1.2 Other Conditions

Coagulopathy

a. Patients must not have had deep venous thrombosis (including pulmonary embolism) within the last three months prior to study enrollment.
b. Patients on full-dose anticoagulants (*e.g.*, warfarin) with PT INR > 1.5 are eligible if both of these criteria are met:
    - The patient has an in-range INR (usually between 2 and 3) on a stable dose of oral anticoagulant or on a stable dose of low molecular weight heparin.

- The patient has no active bleeding or pathological condition that carries a high risk of bleeding (e.g., tumor involving major vessels or known varices).

Cardiovascular Disease or Hypertension

a. Patients must not have had recent (within 6 months) arterial thromboembolic events, including transient ischemic attack (TIA) or cerebrovascular accident (CVA)
b. Patients must not have a history of myocardial infarction, severe or unstable angina, or peripheral vascular disease (Unlikely in the patient population being studied)
c. Hypertension must be well controlled on stable doses of medication for at least two weeks prior to enrollment.

4.1.3 Concomitant Therapy

a. Growth factor(s): Must not have received G-CSF within 1 week or PEG G-CSF within 2 weeks of entry onto this study. Growth factors may not be administered during the course of this study except as described in the treatment section

b. Study Specific: Patients may not receive any other chemotherapy or immunomodulating agents, while enrolled on study. Radiation therapy may not be given during the first two courses of chemotherapy. After two courses, radiation therapy may be given after discussion with the Principal Investigator.

c. Chronic medications: Patients may be on chronic medications (e.g.: steroids, narcotics, anti-epileptics) at the time of enrollment. Investigators should avoid, when possible, increasing or decreasing the doses of these medications while the patient is on study.

d. Enzyme-inducing anticonvulsants or other medications: Patients who are currently taking phenobarbital, phenytoin, carbamazepine, oxcarbazepine (Trileptal), rifampin, voriconazole, itraconazole, ketoconazole, aprepitant (Emend), or St. John’s Wort are not eligible.

4.1.4 Organ Function Requirements

Adequate Bone Marrow Function Defined As:

For patients with solid tumors including status post SCT:

- Peripheral absolute neutrophil count (ANC) $\geq$750/µL
- Platelet count $\geq$75,000/µL (transfusion independent)
- Hemoglobin $\geq$ 8.0 gm/dL (may receive RBC transfusions)

For patients with tumor metastatic to bone marrow who have granulocytopenia, anemia, and/or thrombocytopenia:

- Peripheral absolute neutrophil count (ANC) $\geq$ 500/µL
- Platelet count $\geq$ 50,000/µL (Transfusion independent- should not have received a platelet transfusion in the 5 days prior)
- Hemoglobin $\geq$ 8.0 gm/dL (may receive RBC transfusions)

Hematologic toxicity will not be evaluable in these patients.

Adequate Renal Function Defined As:

- Creatinine clearance or radioisotope GFR $\geq$ 70ml/min/m2 OR

- A serum creatinine based on age as follows:

AGE (years)	Maximum creatinine
<5	0.8
5-10	1
10-15	1.2
>15	1.5

Urine protein should be screened by urine analysis for Urine Protein Creatinine (UPC) ratio. For UPC ratio > 0.5, 24-hour urine protein should be obtained and the level should be < 1,000 mg for patient enrollment.

Note: UPC ratio of spot urine is an estimation of the 24 urine protein excretion – a UPC ratio of 1 is roughly equivalent to a 24-hour urine protein of 1 gm. UPC ratio is calculated using one of the following formulae:

- [urine protein]/[urine creatinine] – if both protein and creatinine are reported in mg/dL
- [(urine protein) x 0.088]/[urine creatinine] – if urine creatinine is reported in mmol/L

Adequate Liver Function Defined As:

-Total bilirubin $\leq$ 1.5 x upper limit of normal for age,

-SGPT (ALT) $\leq$ 5 x upper limit of normal for age.

Adequate Cardiac Function Defined As:

-Shortening fraction of $\geq$ 28% by echocardiogram and qualitatively normal left ventricular function, or

-Ejection fraction of $\geq$ 55% by MUGA.

No prior history of cardiac failure or dysfunction attributed to anthracylines.

4.2 Exclusion Criteria

- Patients who have received bevazucimab and/or Irinotecan previously are ineligible. Non brain tumor patients who have previously received Temozolomide are ineligible.

- Pregnancy or Breast-Feeding: Pregnant patients are ineligible for this study due to the known teratogenic effects of the cytotoxic agents. Pregnancy tests must be obtained in females of childbearing potential prior to enrollment.

- Lactating women must agree not to breast-feed.

- Males or females of reproductive age may not participate unless they have agreed to use an effective contraceptive method.

- Patients Who Have an Uncontrolled Infection will not be eligible for enrollment until all infections are under control.

- Clinically Significant Unrelated Systemic Illness: Patients with serious infections or significant pulmonary, hepatic, renal, or other end-organ dysfunction which in the judgment of the Principal or Co-Investigators would compromise the patient's ability to tolerate prescribed chemotherapy or are likely to interfere with the study procedures or results will not be eligible.

5.0 Treatment Overview:

5.1 Drug Administration

Dosing is based on body surface area. For administration of irinotecan, body surface area should not exceed 2 square meters.

5.1.1 Administration Schedule for VIT-B(Cycles 1-12)

The administration schedule below describes 1 cycle of VIT-B and should be repeated 11 times. Each cycle is 21 days in duration.

The drug doses for VIT will be based on the patient's body surface area (BSA).

Criteria to start each cycle: ANC ≥ 750 /μL and platelet count ≥ 75,000/μL. The ANC often falls after discontinuing myeloid growth factor support (filgrastim). If the ANC has risen to ≥ 750/μL after the nadir but then falls to < 750/ μL, the next cycle, can be given despite ANC < 750/μL.

5.1.1.1 Temozolomide (TEM): P.O once daily

Always administer Temozolomide at least ONE hour before irinotecan

Days: 1-5

Dose: 100mg/m²/dose. Round to the closest 5 mg. See Appendix II for a dosing table.

Administer with a glass of water preferably on an empty stomach to decrease nausea and vomiting. Administer dose at approximately the same time each day. Guidelines for administration of oral Temozolomide to patients who are unable to swallow capsules are provided in the drug monograph (preparation of suspension) and Appendix III. Antiemetics should be given 30 minutes prior to the Temozolomide dose. If emesis occurs within 10 minutes of taking a given dose, then the dose may be repeated once.

5.1.1.2 Bevacizumab (BEVA): IV infusion

Day: 1.

Dose: Bevacizumab 15 mg/kg

Initial infusion time should be over 90 minutes. If there is no reaction following the first administration, it will be given over 60 minutes in the second administration and, if tolerated, over 30 minutes for all subsequent doses

Bevacizumab is incompatible with D_5W (the drug is inactivated).

Patients may not have a planned major surgical procedure (such as resection of recurrent disease) within 28 days of Bevacizumab. Minor surgical procedures (biopsies) need to have occurred > 7 days or wound healed, whichever occurs later, prior to Bevacizumab. For other surgical procedures (such as a line placement) Bevacizumab should be postponed for 24 hours after the procedure.

5.1.1.3 Vincristine (VCR): IV push over one minute

Days: 1, 8

Dose: 1.5 mg/m^2/dose. (**Maximum dose 2 mg**).

Avoid extravasation. Administration through a central line is recommended

5.1.1.4 Irinotecan (IRN): IV over 90 minutes

Days: 1-5

Dose: Level -1=20 mg/m^2
Level 1=30 mg/m^2
Level 1.5=40 mg/m^2
Level 2 =50 mg/m^2

Dose level will be determined at the time of study enrollment. No intra-patient dose escalation is allowed.

5.1.2 Myeloid Growth Factor: SubQ

Filgrastim: (Neupogen) 5 mcg/kg SQ daily starting on Day 6 (at least 24 hours after completion of previous dose of chemotherapy) and continued until ANC ≥ 2000/µL post nadir. Discontinue at least 24 hours prior to next cycle of chemotherapy. Maximum dose of GCSF administered should not exceed 300 micrograms per dose.

PEG Filgrastim: (Neulasta) 100 mcg/kg SQ X 1 24-36 hrs following the last dose of Irinotecan. Max dose: 6mg. If the treating physician chooses to not administer filgrastim or PEG filgrastrim 24-48 hours following the last dose of irinotecan, filgrastrim must be started if the ANC<1000/cmm at anytime during the cycle and continued until the ANC>2000/cmm.

See Section 6.0 for Dose Modifications based on Toxicities.

5.1.3 If surgery is indicated to treat recurrent or progressive tumor, it should be performed no sooner than 28 days after the most recent Bevacizumab dose. Bevacizumab should not be reinstituted sooner than 28 days after surgery. If there are significant postoperative wound complications Bevacizumab should be withheld for a further 3 weeks at investigator discretion. Elective surgery should not be performed during the first two courses of chemotherapy.

5.1.4 If radiation is planned to treat recurrent or progressive disease, radiation should not be performed during the first two courses of chemotherapy. Subsequently, radiation therapy is allowed. Treating physicians must be aware agents used on this study are known to increase the toxicity of radiation. Bevacizumab should be held for significant radiation induced complications and may be reinstituted after 3 weeks at investigator discretion.

5.2 Definition of Course:

A course will be considered 21-days from the start of the chemotherapy on day 1 or until the time of hematopoietic recovery (ANC > 2000/cmm post nadir after growth factor administration or ANC > 750/cmm if no myeloid growth factor is used) and platelet count > 75,000.cmm-(without having received a platelet transfusion in the prior 5 days) or whichever is longer. The interval between each course should not exceed 35 days.

5.3 Criteria for Retreatment

A course may be repeated no earlier than every 21 days if the patient has at least stable disease and has recovered from the prior course of therapy to laboratory parameters defined in the eligibility criteria. This is in addition to all non-hematological, non-dose limiting toxicities returning to grade I level. The duration between courses should not exceed 35 days. (ANC falling to <750/μL after myeloid growth factor has been discontinued is an exception). Patients with DLT who have no evidence of progressive disease may continue on protocol therapy at the lower dose level as long as all toxicities have returned to baseline or ≤ grade 1.

5.4 Dose Escalation

5.4.1 Dose Levels:

Dose Level 1	Irinotecan 30 mg/m^2/day IV on day 1,2,3,4 and 5 (maximum dose 60mg/day)<br>Vincristine 1.5mg/m^2 (2mg max dose) IV on day 1,8<br>Temozolomide 100 mg/m^2 PO on day 1,2,3,4 and 5<br>Bevacizumab 15mg/kg IV on day 1
Dose Level 1.5*	Irinotecan 40 mg/m^2/day IV on day 1,2,3,4 and 5 (maximum dose 80mg/day)<br>Vincristine 1.5mg/m^2 (2mg max dose) IV on day 1,8<br>Temozolomide 100 mg/m^2 PO on day 1,2,3,4 and 5<br>Bevacizumab 15mg/kg IV on day 1

Dose Level 2:	Irinotecan 50 mg/m^2/day IV on day 1,2,3,4 and 5 (maximum dose 100mg/day)<br>Vincristine 1.5mg/m^2 (2mg max dose) IV on day 1,8<br>Temozolomide 100 mg/m^2 PO on day 1,2,3,4 and 5<br>Bevacizumab 15mg/kg IV on day 1
Dose Level -1	Irinotecan 20 mg/m^2/day IV on day 1,2,3,4 and 5 (maximum dose 40mg/day)<br>Vincristine 1.5mg/m^2 (2mg max dose) IV on day 1,8<br>Temozolomide 100 mg/m^2 PO on day 1,2,3,4 and 5<br>Bevacizumab 15mg/kg IV on day 1

1. Cycles will be repeated every 21 days
2. Filgrastim 5 mcg/kg SQ once daily, starting 24-48 hrs after last dose of Irinotecan until ANC> 2000/cmm following nadir or PEG Filgrastim 100mcg/kg SQ X 1 dose 24-48 hrs following last dose of Irinotecan in each cycle. . If filgrastrim or PEG filgrastrim is not given 24-48 hrs after the last dose of irinotecan, filgrastrim must be started if the ANC<1000/cmm at anytime during the cycle and continued until the ANC>2000/cmm
3. Maximum number of cycles = 12

*** Dose escalation will proceed from dose level 1 to dose level 2 and deescalate to dose level 1.5 if DLT is observed on dose level 2.**

Dose escalation will not be continued beyond those dose levels listed above.

5.4.2 Intra-Patient Escalation:

There will be no intra-patient escalation.

5.5 Definitions of Dose-Limiting Toxicity

Dose limiting toxicities are toxicities that occur in the first two courses of therapy, that are possibly, probably or definitely attributable either to Vincristine, Irinotecan, Temozolomide, Bevacizumab or their combination as defined below, with specific exceptions as enumerated.

Toxicity will be graded using the CTCAE criteria, version 4.0. A copy of the CTCAE can be down loaded from the CTEP home page (http://ctep.cancer.gov).

Dose limiting hematologic and non-hematologic toxicities are defined separately.

5.5.1 Non-hematologic dose-limiting toxicity:

1. Any Grade 3 or Grade 4 non-hematological toxicity possibly, probably or definitely attributable to the investigational drug with the specific exclusion of:

    a. Grade 3 nausea, vomiting or dehydration

   b. Grade 3 diarrhea < 3 days , Grade 3 diarrhea regardless of duration if patient did not receive appropriate Supportive care (cefixime, loperamide, or atropine as outlined in Section 7.5)
   c. Grade 3 transaminase (AST/ALT) elevation that returns to Grade ≤ 1 or baseline prior to the time for the next treatment course
   d. Grade 3 GGT
   e. Grade 3 fever, febrile neutropenia, or infection
   f. Grade 3 electrolyte abnormalities (Na, K, Cl, CO2, Ca, Mg, Phosphate) that improve to ≤ Grade 2 within 7 days, with or without supplements
   g. Vincristine-related neuropathy will NOT be considered DLT for the purposes of this study.
   h. Grade 3 catheter related venous thrombosis
2. Grade 2 arterial thrombosis
3. Grade 2 pulmonary/CNS hemorrhage

5.5.2 Hematologic dose limiting toxicity:

Hematologic dose limiting toxicity is defined as:

- Grade IV neutropenia or Grade IV thrombocytopenia lasting > 14 days following the combination Vincristine, Irinotecan, Temozolomide and Bevacizumab chemotherapy.
- Hematologic toxicity that causes a delay of ≥ 14 days beyond the planned interval between treatment courses.

Suspected DLT requires a telephone call to the Principal Investigator or Co investigators as soon as suspected.

The requirement is that DLT are reported immediately to the research coordinator via DLT- reporting CRF in addition to a telephone call to the PI or Co- PI. The CRC, statistician and PI will immediately convene to review the DLT and determine whether the study should continue or be suspended.

5.6 Concurrent Therapies

Appropriate antibiotics, blood products, antiemetics, fluids, electrolytes and general supportive care are to be used as necessary. Other concurrent cancer therapy, including chemotherapy, radiation therapy*, immunotherapy, or biologic therapy cannot be administered to study patients. If these treatments are administered the patient will be removed from study. (* Palliative or therapeutic radiation may be administered following recovery from at least 2 courses of chemotherapy after discussion with the Principal Investigator or Co- Investigators).

5.7 Investigational agents

No other investigational agents may be given while the patient is on study.

6.0 Dose Modifications for Toxicity:

(NCI Common Terminology Criteria for Adverse Events [CTCAE v4.0])

6.1 Dose Modifications/Delays Guidelines for Bevacizumab

Note 1: There will be no dose reduction for Bevacizumab. Treatment should be interrupted or discontinued for certain adverse events, as described below

Treatment Modification for Bevacizumab-Related Adverse Events

Event	CTCAE.v3.0 Grade	Action to be Taken
**Allergic reactions,**<br><br>**or**<br><br>**Acute infusional reactions/ cytokine release syndrome**	Grade 1-2	If infusion-related or allergic reactions occur, premeds should be given with the next dose, and infusion time may not be reduced for the subsequent infusion. **Follow the guidelines in the Section 9.4 for Bevacizumab administration**
	Grade 3	**B**evacizumab infusion should be stopped and not restarted on the same day. At the physicians' discretion, Bevacizumab may be permanently discontinued or re-instituted with premeds and at a rate of 90±15 min a minimum of 24 hours following the initial reaction and before the completion of the last dose of irinotecan for that course. **If Bevacizumab is re-instituted, the patient should be closely monitored for duration comparable to or longer than the duration of the previous reactions**.
	Grade 4	Discontinue Bevacizumab
**Arterial Thrombosis**<br>- Cardiac ischemia/ infarction<br>- CNS ischemia (TIA, CVA)<br>- any peripheral or visceral arterial ischemia/thrombosis	Grade 2 (if new or worsened since Bevacizumab therapy)	Discontinue Bevacizumab.
	Grade 3-4	Discontinue Bevacizumab
**Venous Thrombosis***<br>*for grade 3 catheter related thrombosis the catheter will be removed and Bevacizumab continued as per schedule	Grade 3<br>(non catheter related)	▪ Hold Bevacizumab treatment. If the planned duration of full-dose anticoagulation is <2 weeks, Bevacizumab should be held until the full-dose anticoagulation period is over.<br>▪ If the planned duration of full-dose anticoagulation is > 2 weeks, Bevacizumab may be resumed during the period of full-dose anticoagulation **IF** <u>all</u> of the criteria below are met:<br>− The subject must have an in-range INR (usually 2-3) on a stable dose of warfarin or on stable dose of heparin prior to restarting Bevacizumab.<br>− The subject must not have pathological conditions that carry high risk of bleeding (e.g. tumor involving major vessels or other conditions)<br>− The subject must not have had hemorrhagic events while on study<br>If thromboemboli worsen/recur upon resumption of study therapy, discontinue Bevacizumab
	Grade 4<br>(symptomatic)	Discontinue Bevacizumab
**Hypertension**<br>**Current CTCAE definitions used by CTEP:**	[Treat with anti-hypertensive medication as needed. The goal of BP control should be consistent with general medical practice]	

Event	CTCAE.v3.0 Grade	Action to be Taken
**Use age and gender appropriate normal values >95th percentile ULN for pediatric patients.**	Grade 1: Asymptomatic, transient (<24 hrs) BP increase >ULN; intervention not indicated	Consider increased BP monitoring
	Grade 2 Recurrent or persistent (≥24 hrs) BP >ULN; monotherapy may be indicated	Begin anti-hypertensive therapy and continue Bevacizumab
	Grade 3- requiring more than one drug or more intensive therapy than previously	Discontinue Bevacizumab
	Grade 4: life threatening (e.g. hypertensive crisis)	Discontinue Bevacizumab.
**Congestive Heart Failure**	Grade 3 (symptomatic)	Discontinue Bevacizumab
	Grade 4	Discontinue Bevacizumab
**Proteinuria**	[Proteinuria should be monitored by urine analysis for urine protein creatinine (UPC) ratio prior to every other dose of Bevacizumab]	
	UPC ratio < 3.5	Continue Bevacizumab.
	UPC ratio ≥ 3.5	Discontinue Bevacizumab.
	Grade 4 or nephrotic syndrome	Discontinue Bevacizumab.
**Hemorrhage (CNS or pulmonary)**	Grade 2-4	Discontinue Bevacizumab
**Hemorrhage (non-CNS; non-pulmonary)**	Grade 3	Discontinue Bevacizumab
	Grade 4	Discontinue Bevacizumab
**RPLS** (Reversible Posterior Leukoencephalopathy syndrome **or PRES** (Posterior Reversible Encephalopathy Syndrome)		• **Hold Bevacizumab in patients with symptoms/signs suggestive of RPLS; subsequent management should include MRI scans and control of HTN.**<br>• **Discontinue Bevacizumab upon diagnosis of RPLS.**
**Wound dehiscence** requiring medical or surgical intervention		**Discontinue Bevacizumab**
**GI perforation, GI leak or fistula**		Discontinue Bevacizumab
**Bowel obstruction**	Grade 3-4	• Hold Bevacizumab until complete resolution<br>• If surgery is required, patient may restart Bevacizumab after full recovery from surgery, and at investigator's discretion adhering to the guidelines in Section 5.1.3.
**Other Unspecified Bevacizumab-related AEs** (except controlled nausea/vomiting).	Grade 3	• Hold Bevacizumab until symptoms resolve to ≤ Grade 1
	Grade 4	• Discontinue Bevacizumab<br>• **Upon consultation with the study chair,** resumption of Bevacizumab may be considered if a patient is benefiting from therapy, and the Grade 4 toxicity is transient, has recovered to ≤ Grade 1 and unlikely to recur with

Event	CTCAE.v3.0 Grade	Action to be Taken
		retreatment.

6.2 Other Modifications

6.2.1 Hyperbilirubinemia

If the total bilirubin is 1.5-3 X ULN (grade 2) then hold Vincristine dose for one week. When due next, give full dose if resolved to Grade 1 or less, 50 % of dose if still grade 2; if the total bilirubin is >3 X ULN (grade 3 or 4) then hold Vincristine dose until resolved to Grade 1 or less and dose as above. .

6.2.2 Vincristine Neuropathy

Grade 1 and 2 neurotoxicity requires no dose modification. For Grades 3 and 4 (interfering with activities of daily living) hold until symptoms decrease to Grade 1 (present on exam/testing but not symptomatic) or less and resume at 50% dose. Increases to 75% and full dose should be considered at the start of each Vincristine containing cycle based on patients' symptoms.

Anticipate autonomic neuropathy resulting in constipation. Laxatives and/or stool softeners should be used preemptively during Vincristine containing cycles. If severe paralytic ileus occurs Vincristine should be stopped until normal bowel movements are re-established and then resumed at 50% dose. Mild to moderate constipation (< 4days) is not an indication for interrupting Vincristine.

While Vincristine may be associated with jaw pain this should be treated with analgesics and should not require dose modification.

6.2.3 Renal toxicity

Vincristine and irinotecan are not dose adjusted for renal dysfunction. Caution should be exercised when Temozolomide is administered to patients with severe renal impairment (Creatinine clearance<40ml/min/1.73m2.)

6.2.4 Hematopoietic toxicity

6.2.4.1 Irinotecan and Temozolomide

Prescribe myeloid growth factor routinely as recommended in the treatment plan. If at the time of scheduled therapy the absolute neutrophil count (ANC: PMNS, bands) is < 750/µL on myeloid growth factor, or if the platelet count is < 75,000/µL, further therapy should be delayed until ANC is ≥ 2000/µL on growth factor and platelets ≥ 75,000/µL. If the ANC is < 750µL on myeloid growth factor or platelets < 75,000/µL more than 14 days beyond the time for the next treatment reduce the treatment to the next lower dose level as long as the patient continues to have responsive disease. If similar delays occur, secondary to hematopoietic toxicity with subsequent cycles continue to decrease to the next lower dose level. If already at dose level -1, then decrease Temozolomide dose by 25%.

The ANC often falls after discontinuing myeloid growth factor support (filgrastim). If the ANC has risen to ≥ 750/µL after the nadir but then falls the next cycle can be given despite ANC< 750/µL.

6.2.4.2 Vincristine

No dose adjustment for Vincristine is indicated for hematopoietic toxicity.

6.2.5 Febrile neutropenia

Twenty-five percent dose reductions should also take place in the event of life threatening infection (e.g. septic shock, multiple documented episodes of gram negative sepsis). Uncomplicated febrile neutropenia is not a reason to dose reduce.

6.2.6 Gastrointestinal Toxicity secondary to irinotecan

Patients will receive prophylactic antibiotics and anti-diarrheals to prevent irinotecan induced diarrhea, for detailed information please refer to section 7.0. If patients continue to experience Grade 4 diarrhea or grade 3 diarrhea lasting >3 days despite maximum supportive care measures with prophylactic antibiotics and anti-diarrheals, subsequent irinotecan doses should be reduced to the lower dose level.

6.2.7 Surgical procedures

Patients may not have had a planned major surgical procedure (such as resection of recurrent disease) within 28 days of initiating Bevacizumab. If major surgery for local control is planned during study therapy for a patient on Bevacizumab, then the Bevacizumab should be held for one cycle prior and for 28 days post definitive resection, unless the patient has had a significant post-operative wound complication that in the opinion of the investigator would preclude Bevacizumab administration. In this clinical situation post-operative Bevacizumab should be withheld for longer. These restrictions are due to concerns of delayed wound healing and hemorrhage. Minor surgical procedures (biopsies) need to either have healed or occurred > 7 days prior to initiating Bevacizumab. For other surgical procedures (such as a line placement) initial and subsequent doses of Bevacizumab should be postponed for at least 24 hours after the procedure.

6.2.8 Radiation therapy

No radiation therapy is allowed during the first two cycles of chemotherapy. If radiation therapy is used for treating target lesions, those patients will not be eligible for response evaluation. Radiation therapy should be held for severe chemotherapy related complications at the discretion of the treating oncologist.

7.0 Supportive Care:

General

Appropriate antibiotics, blood product support, and general supportive care measures will be used as indicated according to institutional guidelines and standard of care. Prophylaxis against Pneumocystis jiroveci pneumonia is recommended in all patients. If trimethoprim-sulfamethoxazole is used for prophylaxis, it should be administered on two consecutive days at a dose equivalent to 5 mg/kg of trimethoprim divided BID.

Diarrhea secondary to Irinotecan:

Patients are to receive anti-diarrheals and prophylactic antibiotics (Cefpodoxime or Cefixime) per the supportive care guidelines below.

Patients who have the onset of diarrhea during the irinotecan infusion or in the several hours following completion of the irinotecan infusion should receive a dose of atropine (suggested dose 0.01 mg/kg IV, maximum dose 0.4 mg). Each family should be instructed to have antidiarrheal medication available and begin treatment at the first episode of poorly formed or loose stools or the earliest onset of bowel movements more frequent than normally expected for the patient. Patients should also be instructed to contact their physician if any diarrhea occurs.

Loperamide dosing recommendations for late diarrhea which occurs 8 hours after irinotecan (based on body weight):

Under 13 kg: Take 0.5 mg after the first loose bowel movement, followed by 0.5 mg every 3 hours. During the night, the patient may take 0.5 mg every 4 hours. Do not exceed 4 mg per day.

From 13 kg to less than 20 kg: Take 1 mg after the first loose bowel movement, followed by 1 mg every 4 hours. Do not exceed 6 mg per day.

From 20 kg to less than 30 kg: Take 2 mg after the first loose bowel movement, followed by 1 mg every 3 hours. During the night, the patient may take 2 mg every 4 hours. Do not exceed 8 mg per day.

From 30 kg to less than 43 kg: Take 2 mg after the first loose bowel movement, followed by 1 mg every 2 hours. During the night, the patient may take 2 mg every 4 hours. Do not exceed 12 mg per day.

->12 years old and adults: Take 4 mg after the first loose bowel movement, followed by 2 mg after each loose stool. Do not exceed 16 mg per day.

High dose loperamide (adults) 2mg every 2 hours

Failure of loperamide to control diarrhea within 24 hours of onset:
Begin subcutaneously or intravenously administered octreotide (Sandostatin®), 1-2 mcg/kg/dose every 12 hours. If needed, the dose may be titrated up to 10 mcg/kg/dose (maximum dose: 500 mcg) every 8 hours.

Antibiotics for GI Toxicities

For patients who develop Grade 3 or 4 gastrointestinal (GI) toxicity (see table below for the indications for antibiotic use) following irinotecan therapy, administration guidelines are provided for Cefpodoxime (Vantin®) and cefixime (Suprax®).

Cefpodoxime: 10 mg/kg/day, divided in 2 oral doses; maximum daily dose 400 mg for children < 12 years and maximum daily dose 800 mg for those ≥ 12 years) **OR**

Cefixime: (8 mg/kg/day as a single daily oral dose; maximum daily dose 400 mg).

The antibiotic should be started 5 days prior to the start of irinotecan therapy only if the patient experienced Grade 3 or 4 colitis, dehydration, diarrhea, abdominal pain, weight loss or vomiting during prior therapy with irinotecan. If it is not feasible to start Cefpodoxime or cefixime 5 days prior to therapy with irinotecan, give at least 1 full day of Cefpodoxime or cefixime prior to the start of irinotecan course.

Indications for Antibiotic Use (Cefpodoxime or Cefixime) for GI Toxicities Due to Irinotecan

Toxicity	Defined as
**Abdominal Pain**	Severe pain, pain or analgesics severely interfering with activities of daily living, disabling
**Colitis (Grade 3 or 4)**	Abdominal pain, fever, change in bowel habits with ileus or peritoneal signs, and radiographic or biopsy documentation of perforation or requiring surgery or toxic megacolon.
**Dehydration**	Requiring IV fluid replacement (sustained), physiologic consequences requiring intensive care, hemodynamic collapse.
**Diarrhea**	Increase of $\geq$ 7 stools/day or incontinence; or need for parenteral support for dehydration, severe increase in loose stool, physiologic consequences requiring intensive care, hemodynamic collapse, or watery stool output compared with pretreatment, interfering with normal activity, physiologic consequences requiring intensive care, hemodynamic collapse.
**Vomiting**	$\geq$ 6 episodes in 24 hours over pretreatment, or need for IV fluids requiring parenteral nutrition, or physiologic consequences requiring intensive care, hemodynamic collapse.
**Weight Loss**	> 20%

Adapted from Perry MC et al., ed. *Companion Handbook to Chemotherapy Source Book.* 2nd ed. Baltimore, MD: Lippinkott, Williams and Wilkins; 2004.

8.0 Required Observations/Material and Data to Be Accessioned

8.1 Clinical and Laboratory Studies and Disease Evaluation.

All entry/eligibility studies must be performed within 1 week prior to entry onto the trial (unless otherwise specified). Imaging studies are required within 2 weeks of study entry. An echocardiogram/MUGA may be done up to a month prior to study enrollment.

STUDIES TO BE OBTAINED	Pre-Study*	Course 1	Course 2	Subsequent Courses	Off Study
History	X	X	X	X	X
Physical Exam (Ht, Wt, BSA, VS)	X	X	X	X	X
Blood Pressure	X	X	X	X	X
Performance Status	X	X			X
CBC, differential, platelets[1]	X	Two times/week	Two times/week	Two times/week[2]	X
PT, PTT, INR, D-dimer	X	X	X	X	
Urine for UPC ratio[3]	X	X	X	X	
BUN/Creatinine/Glucose	X	Weekly	X	X	X
Electrolytes including Ca++, PO_4, Mg++	X	X	X	X	X
SGPT, Alk Phos, Bilirubin	X	Weekly		X	X
Echocardiogram/MUGA	X		End of Course		X
Tumor Disease Evaluation – By CT or MRI[4]	X		End of course	End of 5th and 8th course	X
Nuclear Medicine Scan(s)[5]	X		End of course	End of 5th and 8th course	X
Urinary Catecholamines (Neuroblastoma patients only)	X		End of Course	End of 5th and 8th course	X
Bone marrow aspirate and biopsy[6]	X^	X^		End of 5th and 8th course if positive	X if positive
Growth Plate Evaluation[7]	X		End of Course	End of 5th and 8th course	X
Pregnancy Test[8]	X				

- Pre-study evaluation can be used as the initial evaluation for course 1.

OBTAIN OTHER STUDIES AS NEEDED FOR GOOD PATIENT CARE

1. CBC can be discontinued after hematological recovery
2. In patients who have received peg-filgastrim, CBC can be done once a week from 3rd cycle
3. If UPC (Urine Protein Creatinine) ratio > 0.5, 24-hour urine protein should be < 1,000 mg for enrollment
4. Head CT or MRI at baseline is strongly encouraged at baseline to rule out occult brain metastases for non brain tumor patients.
5. Nuclear medicine scans are only required in patients with evaluable disease but without measurable disease. If initially negative, repeat scans are not required unless clinically indicated.
6. If there is suspected BM disease, a BM aspirate and biopsy must be performed within 4 weeks of enrollment. If positive repeated after the 2nd, 5th and 8th course until clear of tumor cells.
7. If patients are found to have an open tibial growth plate, and then repeat plain AP radiographs of the same tibial growth plate will be obtained at the end of 2nd,5th, 8th course and at the end of the study.
8. Female patients with childbearing potential require a negative pregnancy test prior to starting treatment.

Growth plate evaluation:

Patients receiving Bevacizumab will have a plain AP radiograph of a single tibial growth plate obtained prior to initial treatment. If patients are found to have a closed tibial growth plate, no further radiographs will be required. If patients are found to have an open tibial growth plate, then repeat plain AP radiographs of the same tibial growth plate will be obtained at the end of 2nd, 5th, 8th course and at the end of study. The baseline radiograph will be compared with each radiograph performed during follow-up.

Patients with evidence of growth plate thickening or other changes should have a knee MRI performed to further assess the degree of physeal pathology. Patients with abnormalities by MRI should continue to have radiographs of the same tibial growth plate before odd-numbered cycles.

9.0 Agent Information

9.1 Vincristine Sulfate (Oncovin®, VCR, LCR) NSC #67574

Source and Pharmacology: Vincristine is an alkaloid isolated from Vinca rosea Linn (periwinkle). It binds to tubulin, disrupting microtubules and inducing metaphase arrest. Its serum decay pattern is triphasic. The initial, middle, and terminal half-lives are 5 minutes, 2.3 hours, and 85 hours respectively; however, the range of the terminal half-life in humans is from 19 to 155 hours. The liver is the major excretory organ in humans and animals; about 80% of an injected dose of Vincristine sulfate appears in the feces and 10% to 20% can be found in the urine. The p450 cytochrome involved with Vincristine metabolism is CYP3A4. Within 15 to 30 minutes after injection, over 90% of the drug is distributed from the blood into tissue, where it remains tightly, but not irreversibly bound. It is excreted in the bile and feces. There is poor CSF penetration.

Toxicity:

	**Common**<br>Happens to 21-100 children out of every 100	**Occasional**<br>Happens to 5-20 children out of every 100	**Rare**<br>Happens to < 5 children out of every 100
**Immediate:**<br>Within 1-2 days of receiving drug		Jaw pain; headache	Extravasation (rare) but if occurs = local ulceration; shortness of breath and bronchospasm
**Prompt:**<br>Within 2-3 weeks, prior to the next course	Alopecia, constipation,	Weakness, abdominal pain; mild brief myelosuppression (leucopenia, thrombocytopenia, anemia)	Paralytic ileus; ptosis, diplopia, night blindness; hoarseness; vocal cord paralysis; SIADH, seizure; defective sweating
**Delayed:**<br>Any time later during therapy	Loss of deep tendon reflexes	Peripheral paresthesias including numbness, tingling and pain; clumsiness; wrist drop, foot drop; abnormal gait	Difficulty walking or inability to walk; veno-occlusive disease (in combination); blindness, optic atrophy; urinary tract disorders including bladder atony, dysuria, polyuria, nocturia, urinary retention; autonomic neuropathy with postural hypotension; 8th cranial nerve damage with dizziness, nystagmus, vertigo and hearing loss
**Unknown Frequency**	Fetal toxicities and teratogenic effects of Vincristine (either alone or in combination with other		

**and Timing:**	antineoplastic agents) have been noted in humans. The toxicities include: chromosome abnormalities, malformation, pancytopenia, and low birth weight. It is unknown whether the drug is excreted in breast milk.

Formulation and Stability:
Vincristine is supplied in a vial each ml of which contains Vincristine sulfate, 1 mg (1.08 µmol); mannitol, 100 mg; sterile water for injection; Acetic acid and sodium acetate are added for pH control. The pH of Vincristine Sulfate Injection, USP ranges from 3.5 to 5.5. This product is a sterile solution. Store refrigerated at 2-8˚C or 36-46˚F. Protect from light and retain in carton until time of use.

Do not mix with any IV solutions other than those containing dextrose or saline.

Guidelines for Administration: See the Treatment and Dose Modifications Sections of protocol.

The World Health Organization, the Institute of Safe Medicine Practices (United States) and the Safety and Quality Council (Australia) all support the use of mini bag rather than syringe for the infusion of Vincristine. The delivery of Vincristine via either IV slow push or mini bag is acceptable.

. Vincristine sulfate must be administered via an intact, free-flowing intravenous needle or catheter. Care should be taken to ensure that the needle or catheter is securely within the vein to avoid extravasations during administration. The solution may be injected either directly into a vein or into the tubing of a running intravenous infusion.

When dispensed the container or syringe containing Vincristine must be enclosed in an overwrap bearing the statement “Do not remove covering until moment of injection. Fatal if given intrathecally. For Intravenous use only.”

Supplier: Commercially available from various manufacturers. See package insert for more detailed information.

9.2 Irinotecan ***[CPT-11, Camptothecin-11, 7-ethyl-10-(4-[1-piperidino]-1-piperidino) carbonyloxy-camptothecin), Camptosar®], NSC #616348***

Source and Pharmacology: Irinotecan is a semi synthetic water-soluble analog of camptothecin (a plant alkaloid isolated from *Camptotheca acuminata)*. Irinotecan is a prodrug that requires conversion, by the carboxyl esterase enzyme to the topoisomerase-I inhibitor, SN-38 in order to exert anti-tumor activity. SN-38 is approximately 1000 times more potent than irinotecan. Camptothecins interact specifically with the enzyme topoisomerase I which relieves torsional strain in DNA by inducing reversible single-strand breaks. Irinotecan and its active metabolite SN-38 bind to the topoisomerase I-DNA complex and prevent religation of these single-strand breaks. Current research suggests that the cytotoxicity of irinotecan is due to double-strand DNA damage produced during DNA synthesis when replication enzymes interact with the ternary complex formed by topoisomerase I, DNA, and either irinotecan or SN-38. Renal excretion is a minor route of elimination of irinotecan. The majority of the drug is

metabolized in the liver. SN-38 is conjugated to glucuronic acid and this metabolite has no anti-tumor activity. The extent of conversion of SN-38 to its glucuronide has been inversely correlated with the risk of severe diarrhea, because the other major route of SN-38 excretion is biliary excretion by canalicular multi specific organic anion transporter (cMOAT) which presumably leads to mucosal injury. In addition, APC and NPC are oxidative metabolites of irinotecan dependent on the CYP3A4 isoenzyme. After intravenous infusion of irinotecan in humans, irinotecan plasma concentrations decline in a multi exponential manner, with a mean terminal elimination half-life of about 6 to 12 hours. The mean terminal elimination half-life of the active metabolite SN-38 is about 10 to 20 hours. Irinotecan is 30% to 68% bound to albumin and SN-38 is approximately 95% bound to albumin.

Toxicity:

	**Common**<br>Happens to 21-100 children out of every 100	**Occasional**<br>Happens to 5-20 children out of every 100	**Rare**<br>Happens to < 5 children out of every 100
**Immediate:**<br>Within 1-2 days of receiving drug	Nausea, vomiting, anorexia, fever, asthenia, cholinergic symptoms: (rhinitis, increased salivation, miosis, lacrimation, diaphoresis, flushing, and intestinal hyper peristalsis that can cause abdominal cramping and early diarrhea)	Constipation, headache, diarrhea (L)	Anaphylaxis, dehydration with dizziness & hypotension, bradycardia, dyspnea and cough, disorientation/confusion, somnolence, pain at infusion site
**Prompt:**<br>Within 2-3 weeks, prior to the next course	Neutropenia, alopecia, eosinophilia, elevations in transaminases, alkaline phosphatase, bilirubin, mucositis, infection	Anemia, rash, dyspepsia, thrombocytopenia	Colitis, renal failure (secondary to severe dehydration), thromboembolic events, ileus
**Delayed:**<br>Any time later during therapy			Pneumonitis
**Late:**<br>Any time after completion of treatment			
**Unknown Frequency and Timing**:	Fetal toxicities and teratogenic effects of irinotecan have been noted in animals at doses similar or less than those used in humans. Toxicities include: decreased skeletal ossification, multiple anomalies, low birth weight and increased fetal mortality. It is not known if irinotecan is excreted into breast milk but it is excreted into rat milk.		

(L) Toxicity may also occur later.

Formulation & Stability:

Each ml of irinotecan injection contains 20 mg irinotecan (on the basis of the trihydrate salt); 45 mg sorbitol; and 0.9 mg lactic acid. When necessary, pH has been adjusted to 3.5 (range, 3.0 to 3.8) with sodium hydroxide or hydrochloric acid. Irinotecan is available in single-dose amber glass vials in 40 mg (2 ml) and 100 mg (5 ml). Store at controlled room temperature 15°-30°C (59°-86°F). Protect from light. It is recommended that the vial (and backing/plastic blister) should remain in the carton until the time of use.

Guidelines for Administration:
Irinotecan must be diluted prior to infusion. Irinotecan should be diluted in 5% Dextrose Injection, *USP*, (preferred) or 0.9% Sodium Chloride Injection, *USP*, to a final concentration range of 0.12-2.8 mg/Mr. The solution is physically and chemically stable for up to 24 hours at room temperature (approximately 25°C) and in ambient fluorescent lighting. Solutions diluted in 5% Dextrose Injection, *USP*, and stored at refrigerated temperatures (approximately 2°-8°C), and protected from light are physically and chemically stable for 48 hours. Refrigeration of admixtures using 0.9% Sodium Chloride Injection, *USP*, is not recommended due to a low and sporadic incidence of visible particulates.

Supplier:
Commercially available from various manufacturers. See package insert for more detailed information.

9.3 Temozolomide *(TemodarTM) NSC# 362856*

Source and Pharmacology:
An orally administered alkylating agent, a second generation imidazo tetrazine. A prodrug of MTIC, Temozolomide spontaneously decomposes to MTIC at physiologic pH. Exerts its effect by cross-linking DNA. This is likely a site specific alkylation at the O^6-position of guanine with some effect at the N7 position. Temozolomide reaches its peak concentration in 1 hour. Food reduces the rate and extent of absorption. It has an elimination half-life of 1.13 hr (intraperitoneally) and 1.29 hr (orally) with an oral bioavailability of 0.98. Total apparent body clearance is 100 mL/min/m^2 and plasma elimination half-life is ~ 100 minutes.

	**Common**<br>Happens to 21-100 children out of every 100	**Occasional**<br>Happens to 5-20 children out of every 100	**Rare**<br>Happens to < 5 children out of every 100
**Immediate:**<br>Within 1-2 days of receiving drug	Anorexia, constipation, nausea, vomiting,	Abdominal pain, diarrhea, headache, rash, itching, urinary frequency and/or infection	Convulsions, anaphylaxis, hemi paresis, dizziness, ataxia, confusion, dysphagia, anxiety, thrombo-embolism (L)
**Prompt:**<br>Within 2-3 weeks, prior to next course	Myelosuppression	Mucositis, lethargy, peripheral edema	Prolonged lymphopenia with increased risk of infection or death, amnesia, insomnia, depression, myalgia, diplopia, visual changes
**Delayed:**<br>Anytime later during therapy		Alopecia, hepatotoxicity	
**Late:**<br>Anytime after completion of therapy			Secondary tumors or cancer

(L) Toxicity may also occur later.

Formulation and Stability: 5 mg, 20 mg, 100 mg, 140 mg, 180 mg 250 mg capsules, stored at room temperature.

Guidelines for Administration: See Treatment and Dose Modifications sections of the protocol.

There is a potential for medication errors involving Temodar™ capsules resulting in drug over dosage, which may have been caused by dispensing/taking the wrong number of capsules per day and/or product usage exceeding the prescribed dosing schedule. Temodar™ capsules are available in four different strengths, each a different size, and are color coded according to strength. All capsules are available in 5-count and 20-count packages.

Capsule Strength	COLOR
5 mg	Green Imprint
20 mg	Brown Imprint
100 mg	Blue Imprint
140 mg	Blue Cap
180 mg	Orange Cap
250 mg	Black Imprint

When dispensing, it is extremely important that prescribing and dispensing include clear instructions on which capsules, and how many of each capsule(s) are to be taken per day. Only dispense what is needed for the course, and clearly indicate how many days of dosing the patient will have and how many days are without Temodar™ dosing. When counseling patients, it is important for each patient/parent to understand the number of capsules per day and the number of days that they take Temodar™. It is also important for the patient/parent to understand the number of days that they will be off the medication.

Each strength of Temodar™ must be dispensed in a separate vial or in its original glass bottle. Based on the dose prescribed, determine the number of each strength of Temodar™ capsules needed for the full course as prescribed by the physician. For example, 275 mg/day for 5 days would be dispensed as five 250-mg capsules, five 20-mg capsules, and five 5-mg capsules. Label each container with the appropriate number of capsules to be taken each day. Dispense to the patient/parent, making sure each container lists the strength (mg) per capsule and that he or she understands to take the appropriate number of capsules of Temodar™ from each bottle or vial to equal the total daily dose prescribed by the physician.

For children unable to swallow the capsules whole, the oral capsules may be formulated into a suspension. To prepare a 10mg/mL suspension triturate the contents of ten 100mg capsules (1000mg), 500mg povidone K-30 and 25mg anhydrous citric acid dissolved in 1.5mL purified water in a glass mortar to form a uniform paste. To the paste add 50 ml of Ora-Plus® by adding a small amount, mixing, and then adding the balance. Transfer to a glass graduated cylinder. Add Ora-Sweet® or Ora-Sweet® SF to a total volume of 100mL

by rinsing the mortar with small amounts of the syrup (Ora-Sweet® or Ora-Sweet® SF). Rinse at least four times. Package in an amber plastic prescription bottle. The packaged suspension is stable for 7 days at room temperature or 60 days in the refrigerator. The suspension should be shaken well before each use. Procedures for proper handling and disposal of cytotoxic drugs should be used when preparing the suspension. (Trissel LA, Yanpin Z, KiintzSE. Temozolomide stability in extemporaneously compounded oral suspensions. *Int J Pharm Compounding* 10:396-9, 2006.)

Alternatively, the capsules can be opened and mixed with apple sauce or juice (see Appendix III in the protocol).

Supplier: Commercially available. See package insert for further information

9.4 Bevacizumab ***(rhuMAb VEGF, Avastin®) NSC# 704865***

Classification: Recombinant humanized monoclonal antibody

Molecular Weight: Approximate molecular weight is 149,000 daltons

Mode of Action: Bevacizumab blocks the binding of vascular endothelial growth factor (VEGF) to its receptors resulting in inhibition of angiogenesis.

Description: Bevacizumab is a recombinant humanized anti-VEGF monoclonal antibody, consisting of 93% human and 7% murine amino acid sequences. The agent is composed of human IgG framework and murine antigen-binding complementarity-determining regions. The estimated half-life of Bevacizumab is approximately 20 days (range 11-50 days). The predicted time to reach steady state was 100 days in 491 patients who received 1 to 20 mg/kg weekly, every 2 weeks, or every 3 weeks. The clearance and the central volume of distribution are higher in males than females. Clearance was higher in those patients with a higher tumor volume.

Toxicity:

	**Common**<br>Happens to 21-100 children out of every 100	**Occasional**<br>Happens to 5-20 children out of every 100	**Rare**<br>Happens to < 5 children out of every 100
**Immediate:**<br>Within 1-2 days of receiving drug	Asthenia, hypertension, nausea, vomiting, anorexia, diarrhea, constipation, cough, pruritis, headache dyspnea	Dyspepsia, fever,<br>chest, back and abdominal pain, dizziness, rhinitis	Infusion reactions: (dyspnea, ↓O_2, wheezing, hypertensive crisis, chest pain, rigors, headache, diaphoresis), syncope, hypotension), flatulence, dry mouth, taste disorder, tearing, rash, urticaria, hives, drowsiness, acidosis
**Prompt:**<br>Within 2-3 weeks,	Epistaxis, proteinuria, infection, myalgia, arthralgia,	Stomatitis, upper respiratory infection, GI hemorrhage,	Intra-abdominal thrombosis (L), DVT, arterial thromboembolic

prior to next course	neutropenia when added to chemotherapy regimens	GI ulcer, alopecia, weight loss, sinusitis, anemia, neutropenia/leucopenia, voice change, depression, UTI, febrile	events (CVA, MI, TIA, angina - higher incidence (8.5%) in older patients > 65 yrs with previous hx), exfoliative dermatitis, skin ulcer, ↑ AST/ALT, ↑ creatinine, hemorrhage (pulmonary, CNS, GU), colitis, GI perforation (L), GI obstruction, hypokalemia, hyperkalemia, hyperbilirubinemia, thrombocytopenia, hyperesthesia, paresthesia, Reversible Posterior Leukoencephalopathy Syndrome (RPLS), Posterior___Reversible Encephalopathy Syndrome (PRES) or similar leukoencephalopathy syndrome, fatal infections when used in combo with chemotherapy regimens
**Delayed:** Any time later during therapy, excluding the above conditions		Nasal-septal perforation	Wound dehiscence, CHF (increased to 14% with **concurrent** anthracycline) (L), nephrotic syndrome, tracheoesophageal fistula in combination with chemo and radiation therapy involving the esophagus, trachea or main bronchus, renal failure, necrotizing fasciitis, osteonecrosis, tumor necrosis leading to pneumothorax
**Unknown Frequency and Timing**:	Bevacizumab has been shown to be teratogenic in rabbits when administered in doses that are two-fold greater than the recommended human dose on a mg/kg basis. Observed effects included decreases in maternal and fetal body weights, an increased number of fetal resorption, and an increased incidence of specific gross and skeletal fetal alterations. Adverse fetal outcomes were observed at all doses tested. Angiogenesis is critical to fetal development and the inhibition of angiogenesis following administration of Bevacizumab is likely to result in adverse effects on pregnancy. It is not known whether Bevacizumab is secreted in human milk. Because human IgG1 is secreted into human milk, the potential for absorption and harm to the infant after ingestion is unknown.		

***(L) Toxicity may also occur later*.**

How Supplied: Bevacizumab is supplied as a clear to slightly opalescent, sterile liquid ready for parenteral administration in two vial sizes:

1. Each 100 mg (25 mg/mL – 4 mL fill) glass vial contains bevacizumab with phosphate, trehalose, polysorbate 20, and Sterile Water for Injection, USP.
2. Each 400 mg (25mg/ml – 16 mL fill) glass vial contains bevacizumab with phosphate, trehalose, polysorbate 20, and Sterile Water for Injection, USP.

Storage: Bevacizumab vials should be stored in the refrigerator at 2-8°C (36-46°F) until use. Bevacizumab vials should be protected from light. Store vials in original carton until ready for use. Do not freeze. Do not shake.

Stability: The sterile single use vials contain no antibacterial preservatives. Therefore, discard vials 8 hours after initial entry.

Preparation: Vials contain no preservatives and are intended for single use only. Dilute the dose volume in 0.9% sodium chloride for injection to a final concentration within the range of 1.4-16.5 mg/mL. Once diluted administer bevacizumab solutions within 8 hours. Bevacizumab is incompatible with Dextrose solutions.

Administration: See Treatment and Dose Modification Sections of the Protocol

Route of Administration: Intravenous

Method of Administration:

To insure complete delivery of bevacizumab, the IV infusion line must be flushed with 0.9% sodium chloride. The following are two recommended methods for flushing the line:

1. When the bevacizumab infusion is complete, while maintaining a closed system, add an additional 50mL of 0.9% sodium chloride for injection to the bevacizumab infusion bag. Continue the infusion until a volume equal to that of the volume contained in the tubing has been administered.
2. Replace the empty bevacizumab infusion bag with a 50mL bag of 0.9% sodium chloride for injection and infuse a volume equal to the volume contained in the tubing.

Please note: the flush is not included in the total recommended infusion times.

Supplier: commercially available

9.5 Myeloid Growth Factors
Filgrastim (Neupogen), or PEG Filgrastim (Neulasta)
Source and Pharmacology
r-metHuG-CSF (produced in E. coli by recombinant DNA technology) stimulates the production of neutrophils in the bone marrow and selected end-cell activation. The 175 amino acid protein (M.W. of 18,800 daltons) differs from the natural protein in that the N-terminal amino acid is a methionine and it is not o-glycosylated. 3.45 mcg to 11.5 mcg of G-CSF administered subcutaneously resulted in a maximum serum concentration of 4 ng/ml to 49 ng/ml within 2 to 8 hours. The elimination half-life is similar for SQ and IV, approximately 3.5 hours.

Formulation and Stability
Supplied as a clear solution in 300 mcg/ml (1 ± 0.6 x 108 U/mg) (1 ml or 1.6 ml) vials. Vials are preservative free and are intended to be single-use vials; do not reuse opened vials. Filgrastim must be stored between 2° and 8°C. Stability has been demonstrated for at least 24 months when stored under these conditions. Boxes of Neupogen® contain an

indicator that turns red when exposed to freezing temperatures; medication should not be used in the event the indicator changes. Do not use if discolored or if there is particulate matter. For IV use, dilute in D5W to concentrations > 15 mcg/ml; G-CSF is incompatible with normal saline. At dilutions from 5 mcg/ml to 14 mcg/ml, add human serum albumin to a final albumin concentration of 2 mg/ml to protect against absorption of the G-CSF to container walls (glass or plastic). Filgrastim, when diluted as described above, is compatible with a number of plastics commonly used in the manufacture of syringes, IV bags, infusion sets, and IV pump cassettes. These include polyvinyl chloride, polyolefin, and polypropylene. Diluted filgrastim should be stored at 2° to 8° C and used within 24 hours. **Do not shake or freeze.**

Guidelines for Administration

Administer once daily, subcutaneously without dilution or if necessary dilute with 5% dextrose in water, preferably to concentrations of 15 mcg/ml or greater for IV administration. Dilutions should be prepared as close to the time of administration as possible (up to 24 hours), since the product is preservative-free. When diluting Filgrastim to 5-14 mcg/ml in D5W, it is necessary at all times to add human serum albumin, to reach a final albumin concentration of 2 mg/ml. The dose of filgrastim is 5 mcg/kg/day (maximum dose 300 mcg). Continue once daily until the ANC is >2000/mm^3 after the nadir has been reached. The dose of PEG-filgrastim is 100 mcg/kg (maximum dose 6 mg) given as a single dose. Myeloid growth factor should be discontinued at least 24 hours prior to receiving Temozolomide.

Supplier: Commercially available:

Toxicities: (See package insert for further information).

	**Common**<br>Happens to 21-100 children out of every 100	**Occasional**<br>Happens to 5-20 children out of every 100	**Rare**<br>Happens to <5 children out of every 100
**Immediate:** Within 1-2 days of receiving drug		Local irritation at the injection site	Allergic reaction, low grade fever
**Prompt:** Within 2-3 weeks, prior to next course		Medullary bone pain, increased alkaline phosphatase, increased lactate dehydrogenase, increased uric acid, thrombocytopenia	Subclinical splenomegaly, exacerbation of pre-existing skin rashes, alopecia
**Delayed:** Anytime later during therapy, excluding the above conditions			Cutaneous
**Late:** Anytime after completion of treatment			

10.0 Criteria for Removal from Protocol Therapy and Off Study Criteria

10.0.1 Criteria for Removal from Protocol Therapy

a. Progressive disease
b. Irreversible dose-limiting toxicity
c. Patient withdrawal from protocol
d. Physician determines it is not in the patient's best interest to continue on protocol therapy.
e. Entry onto another therapeutic study.
f. Diagnosis with a second malignant neoplasm
g. Thirty days after the last dose of the study drugs are given (but note that patients with ongoing toxicity should be followed until the toxicity resolves even if it is longer than 30 days).

Patients who are off protocol therapy are to be followed until they meet the criteria for off study (see below).

10.0.2 Off Study Criteria

a. Death
b. Lost to follow-up
c. Study closure*

* Following completion of protocol therapy vital status and disease status will be followed based on routine clinical visits (not protocol specific visits) for a period of one year after all enrolled subjects have completed therapy. At this time the study will be closed.

11.0 Statistical Considerations

11.0.1 Sample Size and Study Duration

This trial is a phase I evaluation of Irinotecan, Vincristine, Temozolomide and Bevacizumab and G-CSF for hematopoietic support with the starting dose and schedule as stated in the treatment plan section.

It is anticipated that 1-2 patients per month are available for enrollment, of which approximately one half are less than 12 years of age at enrollment. The study will follow a standard 3+3 patient cohort escalation design, with escalation through two dose levels and possible de-escalation to one level below the starting dose level and one level below the second dose level. A minimum of 6 patients and a maximum of 24 patients will therefore be required to establish a recommended phase II dose (RP2D). Patients who do not receive a complete course of treatment for reasons unequivocally unrelated to toxicity are considered not evaluable for DLT evaluation and will be replaced.

11.0.2 Definitions

Evaluable For Toxicity

Dose escalation will not be considered until at least 3 evaluable patients have been entered at the current dose level. For the purpose of dose escalation, a patient who experiences no DLT will be evaluable for toxicity if the patient completes the first 2 courses.

11.0.3 Dose Escalation.
Dose escalations will follow a standard 3-cohort escalation plan.

1. Three patients are studied at the first dose level.
   a. If none of these three patients experience DLT, then the dose is escalated to the next higher level in the three subsequent patients.
   b. If one of three patients experiences DLT at the current dose, then up to three more patients are accrued at the same level.
      I. If none of these three additional patients experience DLT, then the dose is escalated in subsequent patients.
      II. If one or more of these three additional patients experiences DLT, then patient entry at that dose level is stopped, the MTD has been exceeded and dose escalation will be stopped. Three more patients are treated at the next lower dose (unless six patients have already been treated at that prior dose).
   c. If two or more patients experience DLT, then the MTD has been exceeded and dose escalation will be stopped. Three more patients are treated at the next lower dose (unless six patients have already been treated at that prior dose).

The estimated MTD is the highest dose level at which no more than 1/6 patients experience DLT.

If the MTD has been exceeded at the dose level 1, then the subsequent cohort of patients will be treated at dose level –1.
If the MTD has been exceeded at the dose level 2, then the subsequent cohort of patients will be treated at dose level 1.5.

Patients are evaluable for DLTs only during the first two courses of therapy.

11.0.4 Inclusion of Women and Minorities

The study is open to all participants regardless of gender or ethnicity. The small number of patients entered into this trial will obviate any analysis of variation in toxicity profile or response rate with gender or ethnicity.

12.0 Evaluation Criteria

This study will utilize the National Cancer Institute (NCI) CTC Version 4.0.A copy of the NCI-CTC version 4.0 can be downloaded from the CTEP home page (http://ctep.info.nih.gov).Additionally, the toxicities are to be reported on the appropriate data collection forms.

12.0.1 Response Criteria for Non CNS solid tumors:

This study will use the (RECIST) Response Evaluation Criteria in Solid Tumor version 1.1 from the NCI. Response will be assessed using radiographic studies as outlined in section 8.1.

Measurable Disease

The presence of at least one lesion that can be accurately measured in at least one dimension with the longest diameter at least 20 mm. With spiral CT scan, lesions must be at least 10 mm. The investigator will identify up to 5 measurable lesions to be followed for response. Serial measurements of lesions are to be done with CT or MRI. The same method of assessment is to be used to characterize each identified and reported lesion at baseline and during follow-up.

Quantification of Disease Burden

The sum of the longest diameter (LD) for all target lesions will be calculated and reported as the disease measurement.

Complete Response (CR)

Disappearance of all target lesions. If immunocytology is available, no disease must be detected by that methodology.

Partial Response (PR)

At least a 30% decrease in the disease measurement, taking as reference the disease measurement done to confirm measurable disease at study entry.

Progressive Disease (PD)

At least a 20% increase in the disease measurement, taking as reference the smallest disease measurement recorded since the start of treatment; or the appearance of one or more new lesions.

Stable Disease (SD)

Neither sufficient shrinkage to qualify for PR nor sufficient increase to qualify for PD, taking as reference the smallest disease measurement since the treatment started.

Response Assessment

Each patient will be classified according to their “best response” for the purposes of analysis of treatment effect. Best response is determined from the sequence of the objective statutes described above.

Best Response

Two objective status determinations of CR before progression are required for best response of CR. Two determinations of PR or better before progression, but not

qualifying for a CR, are required for a best response of PR. Two determinations of stable/no response or better before progression, but not qualifying as CR or PR are required for a best response of stable/no response; if the first objective status is unknown, only one such determination is required. Patients with an objective status of progression on or before the second evaluations will have a best response of progressive disease. Best response is unknown if the patient does not qualify for a best response of increasing disease and if all objective statuses after the first determination and before progression are unknown.

Sequences of objective statuses with corresponding best response.

1st Status	2nd Status	3rd Status	Best Response
Progression			Increasing Disease
Stable, PR, CR	Progression		Increasing Disease
Unknown	Progression		Increasing Disease
Stable	Stable	Progression	Stable
Stable, Unknown	PR, CR	Progression	Stable
Stable, Unknown	Unknown	Progression	Unknown
PR	PR	Progression	PR
PR	CR	Progression	PR
PR, CR	Unknown	Progression	Unknown
CR	CR	Progression	CR
Unknown	Stable	Progression	Stable

12.0.2 Patients with Evaluable, but not Measurable Disease Only

For patients with evaluable but not measurable disease, only CRs will be considered a response, except for patients with neuroblastoma and germ cell tumors.

For patients with **neuroblastoma** who only had evaluable disease (i.e. limited to positive bone scan, positive MIBG, or bone marrow) with elevated urinary catecholamine(s) (Dopamine, HMMA/VMA or HVA), all of the following criteria must be met for at least 4 weeks to qualify as an **objective response**:

- *MIBG* - must show a decrease in activity if initially abnormal.
- *Urinary catecholamines* - Dopamine, HMMA/VMA and HVA must decrease ≤ 50% of baseline if initially abnormal.
- *Bone Marrow* - if initially positive, must clear on biopsy.

Progression (any of the following)

- Appearance of new areas of malignant disease.
- Worsening of symptoms directly attributable to malignant disease
- In the opinion of the investigator, progression of known sites of disease.
- Sequences of objective statuses with corresponding best responses.

For patients with **germ cell tumors** limited to positive bone scan or disease that is not measurable and with elevated tumor markers, all of the following qualify as an **objective response:**

- Sustained decline in tumor markers between chemotherapy courses
- Pre- course tumor marker level lower than the previous tumor marker level

Progression (any of the following)*-

- -Appearance of new areas of malignant disease.
- -Worsening of symptoms directly attributable to malignant disease.
- -In the opinion of the investigator, progression of known sites of disease.
- -Sequences of objective statuses with corresponding best responses.

* For patients with large tumor burdens and responsive disease, tumor markers may first increase before declining. In this case, response is based on radiographic imaging.

<u>12.0.3 Response Criteria for CNS tumors:</u> [44]

Complete Response (CR)

No evidence of disease at the primary tumor site. Off all steroids with stable or improving neurologic examination.

Partial Response (PR)

A greater than 50% reduction in the product of the greatest tumor diameter and its perpendicular diameter on MRI scan, on a stable or decreasing dose of steroids with a stable or improving neurologic examination.

Minor Response (MR)

A greater than or equal to 25% but less than or equal to 50% reduction in the product of the greatest tumor diameter and its perpendicular diameter on MRI scan, on a stable or decreasing dose of steroids with a stable or improving neurologic examination.

Stable Disease (SD)

A less than 25% reduction in the product of the greatest tumor diameter and its perpendicular diameter on MRI scan, on a stable or decreasing dose of steroids with a stable or improving neurologic examination.

Progressive Disease (PD)

A more than 25% increase in tumor size radiographically or the emergence of new lesions or CSF positivity.

13.0 Adverse Event Reporting Requirements:

Adverse event data collection and reporting which are required as part of every clinical trial, are done to ensure the safety of patients enrolled in the studies as well as those who will enroll in future studies using similar agents. Adverse events are reported in a routine manner at scheduled times during a trial.

Additionally, certain adverse events must be reported in an expedited manner to allow for optimal monitoring of patient safety and care. The following sections provide information about expedited reporting.

Reporting requirements may include the following considerations:

1) The characteristics of the adverse event including the *grade* (severity), the *relationship to the study therapy* (attribution), and the *prior experience* (expectedness) of the adverse event;
2) Whether or not hospitalization or prolongation of hospitalization was associated with the event.

13.0.1 Steps to Determine If an Adverse Event Is To Be Reported In an Expedited Manner

Step 1: Identify the type of adverse event using the NCI Common Terminology Criteria for Adverse Events (CTCAE, V4.0). The CTCAE provides descriptive terminology and a grading scale for each adverse event listed. A copy of the CTCAE can be downloaded from the CTEP home page (http://ctep.cancer.gov/reporting/ctc.html). Additionally, if assistance is needed, the NCI has an Index to the CTCAE that provides help for classifying and locating terms. All appropriate treatment locations should have access to a copy of the CTCAE.

Step 2: Grade the adverse event using the NCI CTCAE.

Step 3: Determine whether the adverse event is related to the protocol therapy

<u>Attribution categories are as follows:</u>

Unrelated, Unlikely, Possible, Probable, and Definite.

Note: This includes all events that occur within 30 days of the last dose of protocol treatment. Any event that occurs more than 30 days after the last dose of treatment and is attributed (possibly, probably, or definitely) to the agent(s) must also be reported according to the instructions above.

Step 4: Determine the prior experience of the adverse event. Expected events are those that have been previously identified as resulting from administration of the agent. An adverse event is considered unexpected, for expedited reporting purposes only, when either the type of event or the severity of the event is not listed in the drug information section of the protocol.

Review the Table below in this section to determine if:
• There are any protocol-specific requirements for expedited reporting of specific adverse events that require special monitoring; and/or
• There are any protocol-specific exceptions to the reporting requirements.

Table A: Phase I Trial Expedited Reporting Requirements for Adverse Events That Occur Within 30 Days[1] of the Last Dose of chemotherapy.

	**Grade 1**	**Grade 2**	**Grade 3**	**Grade 3**		**Grade 3**		**Grades 4 & 5[2]**
	Unexpected and Expected	Unexpected	Expected	Unexpected With Hospitalization	Unexpected Without Hospitalization	Expected With Hospitalization	Expected Without Hospitalization	Unexpected and Expected
Unrelated Unlikely	Not Required	Not Required	Not Required	5 Calendar Days	Not Required	5 Calendar Days	Not Required	24-Hour; 5 Calendar Days
Possible Probable Definite	Not Required	5 Calendar Days	Not Required	24-Hour; 5 Calendar Days	24-Hour; 5 Calendar Days	5 Calendar Days	Not Required	24-Hour; 5 Calendar Days

[1]**Adverse events with attribution of possible, probable, or definite that occur <u>greater</u> than 30 days after the last dose of treatment require reporting as follows:**
24-hour notification (via E-mail or Telephone) to Principal Investigator followed by complete report within 5 calendar days for:

- Grade 3 unexpected events with hospitalization or prolongation of hospitalization
- Grade 4 unexpected events
- Grade 5 expected events and unexpected events

[2]Although 24-hour notification is not required for death clearly related to progressive disease; a full report is required as outlined in the table.

All deaths on study require both routine and expedited reporting regardless of causality. Attribution to treatment or other cause should be provided.

• Any medical event equivalent to CTCAE grade 3, 4, or 5 that precipitates hospitalization (or prolongation of existing hospitalization) must be reported regardless of attribution and designation as expected or unexpected with the exception of any events identified as protocol specific expedited adverse event reporting exclusions.

• Any event that results in persistent or significant disabilities/incapacities, congenital anomalies, or birth defects must be reported if the event occurs following treatment on this study.

• Any death that occurs more than 30 days after the last dose of treatment with an investigational agent which can be attributed (possibly, probably, or definitely) to the agent and is not due to cancer recurrence/progression must be reported.

• Grade 3 or 4 myelosuppression and ≤ Grade 3 febrile neutropenia do not require expedited reporting

13.0.2 When to Report an Event in an Expedited Manner

• Some adverse events require notification **within 24 hours** (refer to Table A) to the Principal Investigator/ Co-Principal Investigator via E-mail or telephone.

• When the adverse event requires expedited reporting, submit the report **within 5 calendar days** of learning of the event.

13.0.3 Definition of Onset and Resolution of Adverse Events

a. If an adverse event occurs more than once in a course (cycle) of therapy only the most severe grade of the event should be reported.
b. If an adverse event progresses through several grades during one course of therapy, only the most severe grade should be reported.
c. The duration of the AE is defined as the duration of the highest (most severe) grade of the Adverse Effects.
d. The resolution date of the AE is defined as the date at which the AE returns to eligibility criteria level (note that the resolution date may therefore be different from the date at which the grade of the AE decreased from its highest grade). If the AE does not return to baseline the resolution date should be recorded as "ongoing."
e. An adverse event that persists from one course to another should only be reported once unless the grade becomes more severe in a subsequent course. An adverse event which resolves and then recurs during a different course, must be reported each course it recurs.
f. Adverse events determined to be reportable must be reported according to the local policy and procedures to the Committee on Clinical Investigations at Childrens Hospital Los Angeles.

13.0.4 Reporting Secondary AML/MDS

All cases of acute myeloid leukemia (AML) and myelodysplastic syndrome (MDS) that occur in patients following their chemotherapy for cancer must be reported immediately to the principal investigator.

14.0 Records and Reporting

14.0.1 Categories or Research Records

Study specific CRF in electronic form will be used. These will be stored in password protected computers accessible only to investigators and study committee members.

When electronic forms are not available paper forms will be used. Research records for this study will be maintained in locked cabinets accessible only to the investigators and study committee members. Subject specific data will be maintained. Data will be retained at least for three years following completion of the research study.

15.0 Data and Safety Monitoring Plan:

The Principal Investigator or designee will be available at all times to answer questions that may rise with patients on the research protocol. Regular meeting will be held with the entire study committee at least once a month, once the first patient is accrued to review study progress. Strict attention will be paid to the conduct of the study according to the protocol since this is a Phase I study. Protocol procedures for safety monitoring have been delineated in the previous sections. The study will continue only if there is unanimous agreement with proceeding after each meeting. In addition, the external Data Safety Monitoring Committee of the Children's Center for Cancer and Blood Diseases will review the conduct of the study at 6 monthly intervals and as needed.

16.0 References:

1. Owellen RJ, Hartke CA, Dickerson RM, Hains FO. Inhibition of tubulin-microtubule polymerization by drugs of the Vinca alkaloid class. *Cancer Res*. 1976; 36(4):1499-1502.

2. Presta LG, Chen H, O'Connor SJ, et al. Humanization of an Anti-Vascular Endothelial Growth Factor Monoclonal Antibody for the Therapy of Solid Tumors and Other Disorders. *Cancer Res*. 1997;57(20):4593-4599.

3. Redinbo MR, Stewart L, Kuhn P, Champoux JJ, Hol WGJ. Crystal Structures of Human Topoisomerase I in Covalent and Noncovalent Complexes with DNA. *Science*. 1998;279(5356):1504-1513.

4. Houghton PJ, Cheshire PJ, Hallman JD, et al. Efficacy of topoisomerase I inhibitors, topotecan and irinotecan, administered at low dose levels in protracted schedules to mice bearing xenografts of human tumors. *Cancer Chemother Pharmacol*. 1995;36(5):393-403.

5. Vassal G, Doz F, Frappaz D, et al. A Phase I Study of Irinotecan As a 3-Week Schedule in Children With Refractory or Recurrent Solid Tumors. *J Clin Oncol*. 2003;21(20):3844-3852.

6. Furman WL, Stewart CF, Poquette CA, et al. Direct Translation of a Protracted Irinotecan Schedule From a Xenograft Model to a Phase I Trial in Children. *J Clin Oncol*. 1999;17(6):1815.

7. Hirota T, Kawai N, Kitagawa S, et al. [Phase I study with irinotecan hydrochloride (CPT-11) for advanced neuroblastoma]. *Gan To Kagaku Ryoho*. 2001;28(13):2049-54.

8. Mugishima H, Matsunaga T, Yagi K, et al. Phase I study of irinotecan in pediatric patients with malignant solid tumors. *J Pediatr Hematol Oncol*. 2002;24(2):94-100.

9. Blaney S, Berg SL, Pratt C, et al. A Phase I Study of Irinotecan in Pediatric Patients: A Pediatric Oncology Group Study. *Clin Cancer Res*. 2001;7(1):32-37.

10. Turner CD, Gururangan S, Eastwood J, et al. Phase II study of irinotecan (CPT-11) in children with high-risk malignant brain tumors: the Duke experience. *Neuro Oncol*. 2002;4(2):102–108.

11. Bomgaars LR, Bernstein M, Krailo M, et al. Phase II Trial of Irinotecan in Children With Refractory Solid Tumors: A Children's Oncology Group Study. *J Clin Oncol*. 2007;25(29):4622-4627.

12. Wagner LM, Crews KR, Iacono LC, et al. Phase I Trial of Temozolomide and Protracted Irinotecan in Pediatric Patients with Refractory Solid Tumors. *Clin Cancer Res*. 2004;10(3):840-848.

13. Wagner LM, McAllister N, Goldsby RE, et al. Temozolomide and intravenous irinotecan for treatment of advanced Ewing sarcoma. *Pediatric Blood & Cancer*. 2007;48(2):132-139.

14. Packer RJ, Jakacki R, Horn M, et al. Objective response of multiply recurrent low-grade gliomas to Bevacizumab and irinotecan. *Pediatric Blood & Cancer*. 2009;52(7):791-795.

15. Wagner LM, Villablanca JG, Stewart CF, et al. Phase I Trial of Oral Irinotecan and Temozolomide for Children With Relapsed High-Risk Neuroblastoma: A New Approach to Neuroblastoma Therapy Consortium Study. *J Clin Oncol*. 2009;27(8):1290-1296.

16. Pappo AS, Lyden E, Breitfeld P, et al. Two Consecutive Phase II Window Trials of Irinotecan Alone or in Combination With Vincristine for the Treatment of Metastatic Rhabdomyosarcoma: The Children's Oncology Group. *J Clin Oncol*. 2007;25(4):362-369.

17. Mascarenhas L. Randomized phase II window study of two schedules of irinotecan (CPT-11) and vincristine (VCR) in rhabdomyosarcoma (RMS) at first relapse/disease progression. *J Clin Oncol*. 2008;26(may 20 suppl;abstr 10013).

18. Wagner LM. Phase I trial and pharmacokinetic study of two schedules of vincristine, oral irinotecan, and temozolomide (VOIT) for children with refractory solid tumors: A Children's Oncology Group Phase I Consortium study. *J Clin Oncol*. 2009;27:15s.

19. Kushner BH, Kramer K, Modak S, Cheung NV. Irinotecan plus temozolomide for relapsed or refractory neuroblastoma. *J. Clin. Oncol*. 2006;24(33):5271-5276.

20. Dvorak HF. Vascular permeability factor/vascular endothelial growth factor: a critical cytokine in tumor angiogenesis and a potential target for diagnosis and therapy. *J Clin Oncol*. 2002;20(21):4368-80.

21. Ferrara N, Davis-Smyth T. The Biology of Vascular Endothelial Growth Factor. *Endocr Rev*. 1997;18(1):4-25.

22. McCrudden KW, Hopkins B, Frischer J, et al. Anti-VEGF antibody in experimental hepatoblastoma: suppression of tumor growth and altered angiogenesis. *J Pediatr Surg*. 2003;38(3):308-14; discussion 308-14.

23. Rowe DH, Huang J, Kayton ML, et al. Anti-VEGF antibody suppresses primary tumor growth and metastasis in an experimental model of Wilms' tumor. *J Pediatr Surg*. 2000;35(1):30-2; discussion 32-3.

24. Kim KJ, Li B, Winer J, et al. Inhibition of vascular endothelial growth factor-induced angiogenesis suppresses tumour growth in vivo. *Nature*. 1993;362(6423):841-4.

25. Rowe DH, Huang J, Li J, et al. Suppression of primary tumor growth in a mouse

model of human neuroblastoma. *J Pediatr Surg*. 2000;35(6):977-81.

26. Gerber HP, Kowalski J, Sherman D, Eberhard DA, Ferrara N. Complete inhibition of rhabdomyosarcoma xenograft growth and neovascularization requires blockade of both tumor and host vascular endothelial growth factor. *Cancer Res*. 2000;60(22):6253-6258.

27. Ferrara N, Hillan KJ, Gerber H, Novotny W. Discovery and development of Bevacizumab, an anti-VEGF antibody for treating cancer. *Nat Rev Drug Discov*. 2004;3(5):391-400.

28. Ryan AM, Eppler DB, Hagler KE, et al. Preclinical safety evaluation of rhuMAbVEGF, an antiangiogenic humanized monoclonal antibody. *Toxicol Pathol*. 1999;27(1):78-86.

29. Yang JC, Haworth L, Sherry RM, et al. A Randomized Trial of Bevacizumab, an Anti-Vascular Endothelial Growth Factor Antibody, for Metastatic Renal Cancer. *N Engl J Med*. 2003;349(5):427-434.

30. Willett CG, Boucher Y, di Tomaso E, et al. Direct evidence that the VEGF-specific antibody Bevacizumab has antivascular effects in human rectal cancer. *Nat. Med*. 2004;10(2):145-147.

31. Lee CG, Heijn M, di Tomaso E, et al. Anti-Vascular endothelial growth factor treatment augments tumor radiation response under normoxic or hypoxic conditions. *Cancer Res*. 2000;60(19):5565-5570.

32. Klement G, Baruchel S, Rak J, et al. Continuous low-dose therapy with vinblastine and VEGF receptor-2 antibody induces sustained tumor regression without overt toxicity. *J. Clin. Invest*. 2000;105(8):R15-24.

33. Hurwitz H, Fehrenbacher L, Novotny W, et al. Bevacizumab plus Irinotecan, Fluorouracil, and Leucovorin for Metastatic Colorectal Cancer. *N Engl J Med*. 2004;350(23):2335-2342.

34. Sandler A, Gray R, Perry MC, et al. Paclitaxel-carboplatin alone or with Bevacizumab for non-small-cell lung cancer. *N. Engl. J. Med*. 2006;355(24):2542-2550.

35. Shah MA, Ramanathan RK, Ilson DH, et al. Multicenter phase II study of irinotecan, cisplatin, and Bevacizumab in patients with metastatic gastric or gastroesophageal junction adenocarcinoma. *J. Clin. Oncol*. 2006;24(33):5201-5206.

36. Bender JLG, Adamson PC, Reid JM, et al. Phase I Trial and Pharmacokinetic Study of Bevacizumab in Pediatric Patients With Refractory Solid Tumors: A Children's Oncology Group Study. *J Clin Oncol*. 2008;26(3):399-405.

37. Reck M, von Pawel J, Zatloukal P, et al. Phase III trial of cisplatin plus gemcitabine with either placebo or Bevacizumab as first-line therapy for nonsquamous non-small-cell lung cancer: AVAil. *J. Clin. Oncol*. 2009;27(8):1227-1234.

38. Vredenburgh JJ, Desjardins A, Herndon JE, et al. Bevacizumab Plus Irinotecan in Recurrent Glioblastoma Multiforme. *J Clin Oncol*. 2007;25(30):4722-4729.

39. Miller K, Wang M, Gralow J, et al. Paclitaxel plus Bevacizumab versus Paclitaxel Alone for Metastatic Breast Cancer. *N Engl J Med*. 2007;357(26):2666-2676.

40. Kabbinavar F, Hurwitz HI, Fehrenbacher L, et al. Phase II, Randomized Trial Comparing Bevacizumab Plus Fluorouracil (FU)/Leucovorin (LV) With FU/LV Alone in Patients With Metastatic Colorectal Cancer. *J Clin Oncol*. 2003;21(1):60-65.

41. Johnson DH, Fehrenbacher L, Novotny WF, et al. Randomized Phase II Trial Comparing Bevacizumab Plus Carboplatin and Paclitaxel With Carboplatin and Paclitaxel Alone in Previously Untreated Locally Advanced or Metastatic Non-Small-Cell Lung Cancer. *J Clin Oncol*. 2004;22(11):2184-2191.

42. Miller KD, Chap LI, Holmes FA, et al. Randomized Phase III Trial of Capecitabine Compared With Bevacizumab Plus Capecitabine in Patients With Previously Treated Metastatic Breast Cancer. *J Clin Oncol*. 2005;23(4):792-799.

43. Giantonio BJ, Catalano PJ, Meropol NJ, et al. Bevacizumab in Combination With Oxaliplatin, Fluorouracil, and Leucovorin (FOLFOX4) for Previously Treated Metastatic Colorectal Cancer: Results From the Eastern Cooperative Oncology Group Study E3200. *J Clin Oncol*. 2007;25(12):1539-1544.

44. Macdonald DR, Cascino TL, Schold SC, Cairncross JG. Response criteria for phase II studies of supratentorial malignant glioma. *J. Clin. Oncol*. 1990;8(7):1277-1280.

APPENDIX I: Performance Status Scales/Scores

Performance Status Criteria<br>Karnofsky and Lansky performance scores are intended to be multiples of 10					
ECOG (Zubrod)		Karnofsky		Lansky	
Score	Description	Score	Description	Score	Description
0	Fully active, able to carry on all pre-disease performance without restriction.	100	Normal, no complaints, no evidence of disease	100	Fully active, normal.
		90	Able to carry on normal activity, minor signs or symptoms of disease.	90	Minor restrictions in physically strenuous activity.
1	Restricted in physically strenuous activity but ambulatory and able to carry out work of a light or sedentary nature, e.g., light housework, office work.	80	Normal activity with effort; some signs or symptoms of disease.	80	Active, but tires more quickly
		70	Cares for self, unable to carry on normal activity or do active work.	70	Both greater restriction of and less time spent in play activity.
2	Ambulatory and capable of all self-care but unable to carry out any work activities. Up and about more than 50% of waking hours	60	Required occasional assistance, but is able to care for most of his/her needs.	60	Up and around, but minimal active play; keeps busy with quieter activities.
		50	Requires considerable assistance and frequent medical care.	50	Gets dressed, but lies around much of the day; no active play, able to participate in all quiet play and activities.
3	Capable of only limited self-care, confined to bed or chair more than 50% of waking hours.	40	Disabled, requires special care and assistance.	40	Mostly in bed; participates in quiet activities.
		30	Severely disabled, hospitalization indicated. Death not imminent.	30	In bed; needs assistance even for quiet play.
4	Completely disabled. Cannot carry on any self-care. Totally confined to bed or chair.	20	Very sick, hospitalization indicated. Death not imminent.	20	Often sleeping; play entirely limited to very passive activities.
		10	Moribund, fatal processes progressing rapidly.	10	No play; does not get out of bed.

*The conversion of the Lansky to ECOG scales is intended for NCI reporting purposes only.

APPENDIX II: Temozolomide Dosing Table-Starting Dose

DOSE= 100mg/$m^{2.}$ Round dose to the nearest 5mg.

BSA (m^2)	Calculated daily dose (mg)	Administered daily dose (mg)
0.28-0.32	28-32	30
0.33-0.37	33-37	35
0.38-0.42	38-42	40
0.43-0.47	43-47	45
0.48-0.52	48-52	50
0.53-0.57	53-57	55
0.58-0.62	58-62	60
0.63-0.67	63-67	65
0.68-0.72	68-72	70
0.73-0.77	73-77	75
0.78-0.82	78-82	80
0.83-0.87	83-87	85
0.88-0.92	88-92	90
0.93-0.97	93-97	95
0.98-1.02	98-102	100
1.03-1.07	103-107	105
1.08-1.12	108-112	110
1.13-1.17	113-117	115
1.18-1.22	118-122	120
1.23-1.27	123-127	125
1.28-1.32	128-132	130
1.33-1.37	133-137	135
1.38-1.42	138-142	140
1.43-1.47	143-147	145
1.48-1.52	148-152	150
1.53-1.57	153-157	155
1.58-1.62	158-162	160
1.63-1.67	163-167	165
1.68-1.72	168-172	170
1.73-1.77	173-177	175
1.78-1.82	178-182	180
1.83-1.87	183-187	185
1.88-1.92	188-192	190
1.93-1.97	193-197	195
1.98-2.02	198-202	200
2.03-2.07	203-207	205
2.08-2.12	208-212	210
2.13-2.17	213-217	215
2.18-2.22	218-222	220
2.23-2.27	223-227	225
2.28-2.32	228-232	230
2.33-2.37	233-237	235
2.38-2.40	238-240	240

APPENDIX III: Instructions for Administration of Temozolomide

(FOR PATIENTS WHO ARE UNABLE TO SWALLOW CAPSULES AND CANNOT OBTAIN A SUSPENSION)

TemodarTM (Temozolomide) is an oral cancer medicine that your child will be taking for treatment of his/her solid tumor. Your child is unable to swallow capsules so the following instructions must be followed for safe administration of this medicine.

- Temodar must be kept in a dark container
- Temodar should be taken the same time everyday
- If your child requires nausea medicine it should be taken prior to the Temodar
- If the dose of Temodar is vomited later than 10 minutes after administration (which is unusual), do not repeat the dose
- If the care giver of the child administering this medicine is pregnant or suspects she is pregnant, she should not handle this medicine

Temodar is an anti-cancer agent, and so special precautions must be taken when handling this medicine. There is potential hazard to anyone who handles this medicine once the protective capsule is opened. Since your child is unable to swallow the capsule you will be required to open the capsules and mix the contents of the capsule in apple sauce or apple juice. This process must be done according to the following guidelines to ensure safe administration of this medicine.

- Find a place that is as free of air flow as possible and is not an area where food is stored or prepared. The work surface should be covered with aluminum foil to reduce exposure to other members of the family.
- Temodar can be mixed in apple sauce or apple juice.
- Place the apple sauce or apple juice in a disposable container.
- Put on disposable gloves.
- Open each capsule and place the powder in a medicine cup.
- Add the whole contents of the medicine cup to either apple sauce or apple juice. The medicine will not dissolve completely if mixing in apple juice so have extra apple juice on hand so you can add it to any remaining powder in the bottom of the cup.
- If you need to have additional juice or apple sauce remove your gloves before touching the main container then place new gloves on before adding the additional juice or apple sauce to the medicine. (You do not want to contaminate the main container with any powder that may be on your gloves)
- Anything that comes into contact with the medicine must be disposable, such as the spoon used for mixing or eating the apple sauce.
- Once all of the medicine is taken throw away the following in the red bag: medicine cup, the container the medicine was mixed in, the cover for the work surface, gloves and anything else that has been in contact with the medicine.

ONCE A COURSE OF MEDICINE IS COMPLETED BRING THE RED BAG WITH YOU TO THE CLINIC SO IT CAN BE DISPOSED OF PROPERLY.
